# Polymorphisms within *DIO2* and *GADD45A* genes increase the risk of liver disease progression in chronic hepatitis b carriers

**DOI:** 10.1038/s41598-023-32753-8

**Published:** 2023-04-14

**Authors:** Magda Rybicka, Eloi R. Verrier, Thomas F. Baumert, Krzysztof Piotr Bielawski

**Affiliations:** 1 Department of Photobiology and Molecular Diagnostics, Intercollegiate Faculty of Biotechnology, University of Gdansk and Medical University of Gdansk, Abrahama 58, 80-307 Gdansk, Poland; 2 Inserm, Institut de Recherche sur les Maladies Virales et Hépatiques UMR_S1110, Université de Strasbourg, 67000 Strasbourg, France; 3 Pôle Hépato-Digestif, Institut Hospitalo-Universitaire, Hôpitaux Universitaires de Strasbourg, 67-000 Strasbourg, France

**Keywords:** Molecular biology, Biomarkers

## Abstract

The study enrolled 284 patients with chronic hepatitis B virus infection. Participants included people with mild fibrotic lesions (32.5%), moderate to severe fibrotic lesions (27.5%), cirrhotic lesions (22%), hepatocellular carcinoma (HCC) in 5%, and people with no fibrotic lesions in 13%. Eleven SNPs within *DIO2*, *PPARG*, *ATF3*, *AKT*, *GADD45A*, and *TBX21* were genotyped by mass spectrometry. The rs225014 TT (*DIO2*) and rs10865710 CC (*PPARG*) genotypes were independently associated with susceptibility to advanced liver fibrosis. However, cirrhosis was more prevalent in individuals with the *GADD45A* rs532446 TT and *ATF3* rs11119982 TT genotypes. In addition, the rs225014 CC variant of *DIO2* was more frequently found in patients with a diagnosis of HCC. These findings suggest that the above SNPs may play a role in HBV-induced liver damage in a Caucasian population.

## Introduction

Hepatitis B is a worldwide disease induced by hepatitis B virus (HBV) infection that affects the liver condition and causes hepatocellular carcinoma development. The natural course of chronic HBV infection (CHB) is long and complicated, with differential underlying changes in liver histology. Patients can shift from a phase with no liver scarring and high viral replication rate to active liver diseases, followed by an inactive hepatitis B phase, and also after years return to the active disease stage. Progression to advanced fibrosis can be rapid, slow, or sporadic. Moreover, liver inflammation, scarring, and even early stage of severe scaring may be reversal after hepatitis B suppression^1–3^. Although the mechanisms by which HBV causes persistent infection are weakly understood, it is known that viral replication itself does not cause liver damage in a short time. Therefore, it is accepted that the individual’s immune response is essential for sustained viral control and the inadequate response is associated with chronic hepatitis B. In fact, 90% of infants whose immune system is not fully matured, become persistently infected when exposed to HBV at birth or in perinatal age. HBV infection results in an acute disease that resolves over time. However, when an individual's immune response fails adults may develop chronic hepatitis B and become more exposed to liver failure development^4^.

Apart from widely known host agents affecting the course of chronic hepatitis B (CHB), such as sex, immune status, and underlying diseases, host genetic background has been extensively studied providing evidence of its role in the susceptibility to HBV persistence, treatment response and the dynamics of liver injury progression to cirrhosis and hepatocellular carcinoma (HCC). The strongest evidence of host genetic factors’ influence on HBV infection outcomes was demonstrated several years ago in twins studies, where higher accordance rates of HBV carriers and antibody titers in response to the HBV vaccine were observed in monozygotic twins in comparison to same-sex dizygotic twins^5,6^. During the past few years, significant progress has been made in genetic and disease-related research due to the development of high-throughput genotyping methods. Since the first implementation of genome-wide association studies (GWAS) on chronic hepatitis B infection in 2009, several single nucleotide polymorphisms (SNPs) have been identified as potential genetic markers influencing the pathogenesis of HBV-related traits^7–13^. However, only a small proportion of SNPs are located in protein-coding regions of the genome, with the vast majority situated within non-coding areas, such as regulatory and intergenic areas, and may thus impact gene regulation^14^.

Moreover, systematic localization of common disease-associated variation has shown that nearly 60% of non-coding GWAS SNPs and other variants are located within DNase I hypersensitive sites (DHSs), which serve key roles in the regulation of gene transcription as markers of cis-regulatory elements (CREs)^15^. Because DHS profiles reflect the occupancy of DNA-binding proteins such as transcription factors (TFs), these loci may alter the transcription factor binding site (TFBS) or induce variation in gene expression^1^. In this study, we have focused on SNPs within TFs and TFBSs of genes associated with the HBV lifecycle, which have been previously associated with multifactorial diseases or traits.

## Results

### Study group characteristics

The study group consisted of 284 chronic hepatitis B (CHB) patients, including individuals with mild fibrosis (92), moderate to severe fibrosis (78), liver cirrhosis—LC (63), hepatocellular carcinoma—HCC (13), and no fibrosis (38) participants. Table 1 summarizes the distribution of variables evaluated at study inclusion. The overall incidence rate of liver cirrhosis and HCC among CHB patients was 22.18% (63/284) and 4.5% (13/284) respectively, with male patients outnumbering females in both subgroups. The mean age of patients with liver cirrhosis was 61 years, and they were significantly older than no fibrosis (p = 0.000146) as well as patients with fibrosis (p = 0.000032). No significant differences in clinical parameters were observed between patients with mild and moderate to severe liver fibrosis. Aspartate aminotransferase (AST) (p = 0.016084) and total cholesterol (TC) (p = 0.014987) levels were higher among individuals with liver cirrhosis versus the no fibrosis group. As well, the prevalence of portal hypertension (HT) and thrombocytopenia (platelet count below 150,000) were much higher in the cirrhotic group.

**Table 1:** Characteristics of chronic hepatitis B patients with liver cirrhosis, hepatic fibrosis, and healthy controls.

Variable	No fibrosis^a^ (n = 38)	Mild fibrosis^b^ (n = 92)	Liver fibrosis^c^ (n = 78)	Liver cirrhosis^d^ (n = 63)	Liver cancer (n = 13)	*p* value^e^
Age, years	49.32 ± 2.47	52 ± 1	52 ± 1	61 ± 2	63 ± 2.66	**0.000146**
Sex, % females	29%	41%	37%	30%	31%	0.897307
Alcohol consumption, %	21%	13%	22%	25%	38%	0.61925
BMI, kg/m^2^	26.39 ± 0.87	26 ± 0.02	25 ± 0.01	27 ± 1	28.38 ± 1.55	0.193066
Diabetes, %	8%	4%	5%	13%	0%	0.45282
HT, %	13%	27%	18.5%	15%	54%	**0.024665**
ALT, IU/L	35.24 ± 3.25	37 ± 2	34 ± 2	46 ± 5	51 ± 5.77	0.068334
AST, IU/L	29.87 ± 2.64	30 ± 1	28 ± 1	43 ± 5	48.77 ± 8.68	**0.016084**
ALB, IU/L	40.66 ± 2.09	41 ± 1	41 ± 1	39 ± 1	38.91 ± 1.65	0.372091
PLT, 10^9^/L	218 ± 14.32	210 ± 5.07	208 ± 6	161 ± 8	159 ± 23	**0.000172**
TC, mmol/L	1.96 ± 0.10	2 ± 0.02	2 ± 0.01	2 ± 0.01	2.09 ± 0.41	**0.014987**
HDL, mmol/L	0.36 ± 0.13	1 ± 0.01	1 ± 0.01	1 ± 0.01	0.67 ± 0.21	0.141507
TG, mmol/L	1 ± 0.12	1 ± 0.13	1 ± 0.01	1 ± 0.02	1.09 ± 0.16	0.130732
GGT, IU/L	71.51 ± 23	50 ± 8	41 ± 5	92 ± 16	179 ± 57	**0.0222304**
HBV DNA, IU/mL	3359 ± 1940	73,773 ± 64,762	34,185 ± 5291	15,015 ± 3787	1.863 ± 1837	** < 0.0001**
HBeAg, % positive	3%	8%	12%	13%	17%	0.119784
HBsAg, IU/ml	12, 769 ± 3582	8947 ± 5022	4.399 ± 2165	5242 ± 2538	7156 ± 5352	**0.043437**
Anti-HBe, % positive	84%	86%	80%	70%	73%	0.143945
Fib-4	1.02 (0.65–1.61)	1.22 (0.8–1.78)	1.15 (0.80–1.77)	2.24 (1.53–3.77)	3.32 (2.07–4.64)	**0.003809**
antyHBs	10.5%	5%	6.48%	14.23%	8%	0.584689

Significant values are in bold.

Unless stated otherwise, data are shown as the mean ± standard error of the mean; ^†^median value (interquartile range); ^a^no scarring (stage 0); ^b^fibrosis stage I; ^c^fibrosis stages II–III; ^d^fibrosis stage IV; ^e^liver cirrhosis vs. no fibrosis; p values less than 0.05 are shown in bold. *BMI* body mass index, *HT* portal hypertension, *ALT* alanine aminotransferase, *AST* aspartate aminotransferase, *ALB* albumin, *PLT* platelet count, *TC* total cholesterol, *HDL* high-density lipoprotein, *TG* triglycerides**, ***GGT* gamma-glutamyl transferase.

DNA samples from all subjects included in the study were successfully analyzed, and high-quality genotyping data was generated for all eleven SNPs. The distribution of genotypes did not follow the Hardy–Weinberg equilibrium (HWE) for the liver fibrosis, cirrhosis, and no fibrosis group except for rs225014, rs2016520, rs4794067 in cirrhotic patients, and rs2016520 in no fibrosis group that was consistent with HWE (p > 0.5). Surprisingly in the HCC group, only rs12031994 (*AKT3*) displayed deviation from HWE. Evaluation of the Linkage Disequilibrium (LD) pattern with the use of the correlation coefficient r2 between pairs of analyzed SNPs showed that all of them were independent (r^2^ < 0.5). The distribution of SNPs genotypes was compared between no fibrosis, fibrosis, cirrhosis, and HCC groups (Table 2). Significant differences in genotype distribution were observed for rs225014 (*DIO2*) and rs4794067 (*TBX21*) between groups of patients affected by different HBV-related liver diseases (Table 2). Rs225014 TT genotype was more common in patients with no fibrosis (52%) in comparison to the cirrhosis group (44%), and its frequency dropped to 8% in patients with HCC. On the other hand, the rs4794067 T allele was more common in patients with more advanced HBV-related liver disease. Moreover, both SNPs within the *GADD45A* gene (rs532446, rs37834688) demonstrated different distributions between cirrhosis and fibrosis groups.

**Table 2:** Genotypic distribution of analyzed SNPs among chronic hepatitis B patients.

SNP ID	Genotype	Genotypic distribution (%)				*p* values			
		Liver cirrhosis (n = 63)	Liver fibrosis (n = 170)	No fibrosis (n = 38)	HCC (n = 13)	Cirrhosis vs. no fibrosis	HCC vs. no fibrosis	Cirrhosis vs. fibrosis	Cirrhosis vs. HCC
*DIO2* rs225014	TT TC CC	28 (44) 28 (44) 7 (12)	36 (21) 45 (26) 89 (52)	20 (52) 7 (19) 11 (29)	1 (8) 7 (54) 5 (38)	**0.010064**	**0.008834**	**0.019885**	**0.010426**
*DIO2* rs225017	TT TA AA	24 (38) 19 (30) 20 (32)	58 (34) 49 (29) 63 (37)	15 (39) 10 (26) 13 (35)	3 (23) 6 (46) 4 (31)	0.91501	0.370214	0.942456	0.466366
*PPARG* rs10865710	CC CG GG	38 (61) 15 (23) 10 (16)	26 (15) 39 (23) 105 (62)	20 (52) 9 (24) 9 (24)	4 (31) 6 (46) 3 (23)	0.60267	0.266059	0.979899	0.136926
*PPARG* rs2016520	TT CT CC	42 (67) 16 (25) 5 (8)	19 (11) 31 (18) 120 (71)	27 (71) 9 (23) 2 (6)	8 (61) 4 (31) 1 (8)	0.843981	0.812338	0.4192	0.922155
*ATF3* rs11119982	CC CT TT	33 (52) 15 (24) 15 (24)	61 (36) 46 (27) 63 (37)	18 (48) 6 (16) 14 (36)	4 (31) 5 (38) 4 (31)	0.324112	0.219991	0.088104	0.346149
*AKT3* rs12031994	TT TC CC	48 (76) 11 (18) 4 (6)	5 (3) 22 (13) 143 (84)	34 (89) 1 (4) 3 (7)	13 (100%) 0 (0%) 0 (0%)	0.082759	0.815154	0.301598	0.484884
*GADD45A* rs532446	TT TC CC	28 (44) 10 (16) 25 (40)	36 (21) 37 (22) 97 (57)	23 (61) 7 (18) 8 (21)	6 (46) 5 (38) 2 (16)	0.147835	0.33893	**0.016846**	0.101074
rs3783468	GG GA AA	26 (42) 18 (28) 19 (30)	56 (33) 39 (23) 75 (44)	14 (37) 10 (26) 14 (37)	7 (54) 3 (23) 3 (23)	0.784711	0.529768	**0.040801**	0.705755
*TBX21* rs4794067	TT TC CC	41 (65) 19 (30) 3 (5)	30 (18) 41 (24) 99 (58)	23 (60) 4 (10) 11 (30)	7 (54) 5 (38) 1 (8)	**0.000871**	**0.044675**	**0.035865**	0.731113

*HCC* hepatocellular carcinoma; *p* values < 0.05 are marked in bold.

### Association of gene polymorphisms with viral and clinical characteristics

We have found significant differences in *DIO2* gene polymorphism between males and females, and rs225014 CC (p = 0.00124) and rs225017 TT (p = 0.00417) genotypes were more common in men. Furthermore, higher HBsAg levels were found in individuals with *AKT3* rs12031994 TT (sex-adjusted TT vs. CC: OR 0.22, 95% CI 0.05–0.75, p = 0.016) genotypes. On the other hand, *AKT3* rs12031994 CC genotype (sex-adjusted TT vs. CC: OR 4.80, 95% CI 1.49–15.43, p = 0.008) was associated with higher AST levels at study inclusion. Additionally, in rs205014 CC (*DIO2*) carriers, the presence of the HBeAg antigen was more common (sex-adjusted TT vs. CC: OR 2.83, 95% CI 1.24–6.47, p = 0.013).

### Gene polymorphisms and liver aminotransferase levels

In a univariate correlation analysis, ALT levels correlated with sex (p = 0.015), thrombocytopenia (p = 0.008), and HBV DNA levels (p = 0.03). Among the SNPs, we have observed significant associations between ALT concentration and the *DIO2*, *GADD45A*, and *AKT3* genotypes (Table 3). The presence of the minor allele at rs204014 and rs12031994, and a major allele at rs205017 and rs532446 were more common in patients with elevated ALT levels.

**Table 3:** Results of logistic regression analyses for elevated ALT risk in chronic hepatitis B patients.

Genotype	Normal levels of serum ALT (n = 216)	Elevated levels of serum ALT (n = 68)	OR (95% CI)	*p* value	Adjusted *p *value^a^
*DIO2* rs225014					
TT	115 (53%)	23 (34%)			
TC	54 (25%)	26 (38%)	2.08 (1.14–3.82)	0.016	0.034
CC	47 (22%)	19 (28%)			
*DIO2* rs225017					
TT	74 (34%)	31 (45%)			
TA	62 (29%)	21 (31%)	0.58 (0.28–0.93)	0.028	0.047
AA	80 (37%)	16 (24%)			
*AKT3* rs12031994					
TT	188 (87%)	50 (73%)			
TC	17 (8%)	12 (18%)	3.10 (1.33–7.23)	0.008	0.010
CC	11 (5%)	6 (9%)			
*GADD45A* rs532446					
TT	110 (51%)	46 (68%)			
TC	45 (21%)	12 (17%)	0.39 (0.20–0.77)	0.006	0.007
CC	61 (28%)	10 (15%)			

*CI* confidence interval, *OR* odds ratio, ^a^*p*-value adjusted for age and sex.

Next, a multivariate regression analysis was used to identify independent predictors of ALT levels in our patients with HBV infection. Serum ALT activity was considered the dependent variable. The results of this analysis showed that thrombocytopenia, rs225014 TT, rs12031994 TT, and rs532446 CC were independently associated with ALT levels (Table 4).

**Table 4:** Final multiple logistic regression model for ALT levels.

Variable	N (%)	OR	95% CI	*p* value
PLT				
> 150 IU/L	244 (84%)	0.35	0.17–0.71	**0.003**
≤ 150 IU/L	40 (16%)			
*DIO2* rs225014				
CC, CT vs. TT	146 (52%) vs. 138 (48%)	0.49	0.27–0.90	**0.021**
*AKT3* rs12031994				
CC, CT vs. TT	46 (16%) vs. 238 (84%)	0.42	0.21–0.82	**0.012**
*GADD45A* rs532446				
TT, TC vs. CC	213 (75%) vs. 71 (25%)	0.45	0.21–0.97	**0.042**

Significant values are in bold.

*CI* confidence interval, *OR* odds ratio; *p* value.

### Genetic polymorphisms and the liver fibrosis progression in chronic hepatitis B

**Figure 1:**
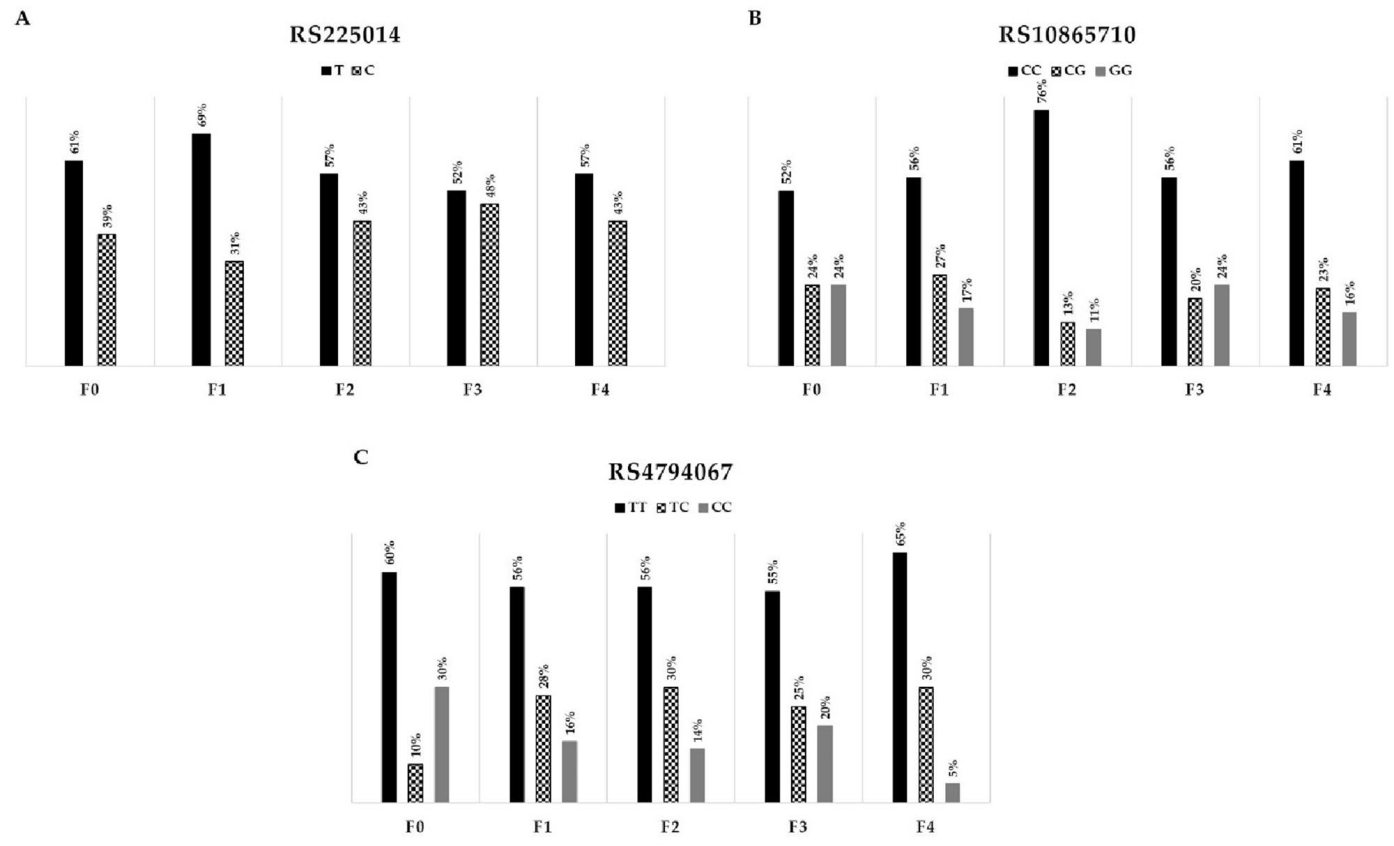
Graphs showing genotype distribution of *DIO2* rs225014 (**A**), *PPARG* rs10865710 (**B**), and *TBX21* rs4794067 (**C**) in patients with different fibrosis scores.

We next assessed the association between analyzed SNPs and liver fibrosis progression. Genotype distribution of the T allele within rs225014 was significantly different in the fibrosis score F0 group when compared to F1 (p = 0.003), F2 (p = 0.012), F3 (p = 0.0002), and F4 (p = 0.0003) patients. Significant differences were also found in genotype occurrence within F0 and F score groups for *PPARG* rs10865710 (p = 0.028), and *TBX21* rs4794067 (p = 0.028, Fig. 1). Also, the *GADD45A* rs532446 TT genotype was more common in the F0 score in comparison to the F4 group.

In further analysis when the cohort was segregated into those with mild (F0–1) versus advanced fibrosis (F2–4), carriage of *DIO2* rs225014 TT and rs225017 AA, and *PPARG* rs10865710 CC genotypes were associated with a significantly increased risk of advanced fibrosis, independent of age and gender (Table S1). In multivariate analyses adopting a dominant model rs225014 TT (*DIO2*) and rs10865710 CC (*PPARG*) and portal hypertension remained an independent predictor of advanced fibrosis (Table 5), whereas *DIO2* rs225017 lost significance (p > 0.05).

**Table 5:** Final multiple logistic regression model for advanced fibrosis (F ≥ 2).

Variable	N (%)	OR	95% CI	*p* value
Portal hypertension				
YES	71 (25%)	0.44	0.21–0.93	**0.032**
NO	213 (75%)			
*DIO2* rs225014				
CC, CT vs. TT	146 (52%) vs. 138 (48%)	0.51	0.29–0.89	**0.018**
*PPARG* rs10865710				
GG,GC vs. CC	117 (41%) vs. 167 (59%)	2	1.18–3.66	**0.010**

Significant values are in bold.

*CI* confidence interval, *OR* odds ratio; *p* value*.*

A corresponding analysis was made to investigate associations between evaluated variabilities and liver cirrhosis. Univariate analyses of variables associated with liver cirrhosis showed significant association observed for age (p < 0.0001), thrombocytopenia (p < 0.0001), total cholesterol levels (p = 0.00014), gamma-glutamyl transferase levels (p < 0.0001), BMI (p = 0.003), alcohol consumption (p = 0.006), ALT level (p 0.0036), AST level (p < 0.0001), *GADD45A* rs532446 (p = 0.033), *ATF3* rs11119982 (p = 0.014), and *TBX24* rs4794067 CC (p = 0.023). Next, in a multiple logistic regression model thrombocytopenias, higher ALT levels, rs532446 TT, and rs11119982 TT remained significant predictors of cirrhosis (Table 6).

**Table 6:** Final multiple logistic regression model for liver cirrhosis.

Variable	N (%)	OR	95% CI	*p* value
PLT				
> 150 IU/L	244 (84%)	0.13	0.05–0.37	**< 0.0001**
≤ 150 IU/L	40 (16%)			
ALT				
> 42 IU/L	57 (20%)	4.79	1.58–14.5	**0.005**
≤ 42 IU/L	227 (80%)			
*ATF3* rs11119982				
CC, CT vs. TT	190 (67%) vs. 94 (33%)	0.42	0.21–0.82	**0.012**
*GADD45A* rs532446				
CC, CT vs. TT	130 (46%) vs. 154 (54%)	0.45	0.21–0.97	**0.042**

Significant values are in bold.

*CI* confidence interval, *OR* odds ratio, *p* value.

HCC was detected in 13 of 284 (4.6%) CHB patients. No association was found between analyzed SNPs and HCC presence. However, different genotypic distribution was found for *DIO2* rs225014 between patients with cirrhosis who have developed primary malignancy of the liver and those without HCC (p = 0.010426). Rs225014 CC variant was identified in 38% of patients with HCC, and 12% of cirrhotic patients without HCC.

### In silico trial results

Using SIFT algorithm substitution at position 92 from T to A was predicted to be tolerated with a score of 0.51. Median sequence conservation was 3.50. SHOPE report showed that the mutant residue is smaller and more hydrophobic than the wild-type residue, and this variant’s MetaRNN score was 2.324709e-05. Furthermore, rs225014 was analyzed by I-Mutant 3.0 and MUpro servers. The free energy change (∆∆G) values were below − 0.5 kcal/mol (∆∆G = − 1.30 for I-Mutatnt 3.0; ∆∆G = − 1.4718185 for MUpro), which indicates that the mutation can largely destabilize the DIO2 protein.

For two SNPs analyzed with RegulomeDB, the predicted rank was 5, which suggested that these SNPs have a minimal probability to affect TF binding and/or DNase peak (Table 7).

**Table 7:** RegulomeDB results for SNPs within selected regions.

SNP ID	Gene	chr:start	Rank	Score
rs225014	*DIO2*	chr14:80203236–80203237	1f	0.55436
rs10865710	*PPARG*	chr3:12353197–12353198	1f	0.07783
rs532446	*GADD45A*	chr1:67686754–67686755	1f	0.70823
rs4794067	*TBX21*	chr17:47731461–47731462	1f	0.55436
rs12031994	*AKT3*	chr1:243917308–243917309	5	0.13454
rs11119982	*ATF3*	chr1:212764843–212764844	5	0.13454

1f—eQTL + TF binding/DNase peak; 5—TF binding/DNase peak; The RegulomeDB probability score ranges from 0 to 1 and the higher it is the more likely to be a regulatory variant.

Remarkably, the highest evidence of regulatory function was shown for rs225014, rs10865710, rs532446, and rs4794067. RegulomeDB revealed that rs10865710 is linked to *PPARG* and *TIMP4* expression, and may likely affect JUN protein binding, as well as falls within NFATC1, NFATC3, NFATC4, and NFAT5 binding motifs. With the same RegulomeDB rank, rs532446 was shown to affect numerous different proteins (Supplementary Table S2) and is localized within ATF4 and PRDM binding motifs. Similarly, rs225014 was demonstrated to affect target gene expression and a variety of protein binding (Supplementary Table S3). Additionally, rs4794067 was shown to have an impact on multiple genes expression (Supplementary Table S4), and influence on EZH2 and CTCF binding.

The histone modification analysis showed that rs10865710, rs532446, and rs4794067 were predicted to locate in enhancer histone marks (liver, endocrine gland, exocrine gland). The key information regarding histone modification analysis restricted to the liver organ is shown in Table 8. More detailed information can be found in Table S5. Furthermore, miRNASNP analysis demonstrated that all SNPs may influence the recognition and targeting of miRNA (Table 9).

**Table 8:** Key histone modification analysis results restricted to liver organ obtained by RegulomeDB.

SNP ID	Chromatin state	Biosample	Classification	Organ
rs10865710	Active enhancer 1	HuH-7	Cell line	Endocrine gland, exocrine gland, liver, epithelium
	Active enhancer 1	HuH-7.5	Cell line	Endocrine gland, exocrine gland, liver, epithelium
	Active enhancer 1	Hepatic stellate cell	Primary cell	Endocrine gland, exocrine gland, liver, connective tissue
rs532446	Active TSS	Liver	Tissue	Endocrine gland, exocrine gland, liver
	Active TSS	Hepatocyte	In vitro differentiated cells	Endocrine gland, exocrine gland, liver, epithelium
	Active TSS	Hepatic stellate cell	Primary cell	Endocrine gland, exocrine gland, liver, connective tissue
	Active TSS	Liver	Tissue	Endocrine gland, exocrine gland, liver
	Active TSS	Liver	Tissue	Endocrine gland, exocrine gland, liver
	Active TSS	HepG2	Cell line	Endocrine gland, exocrine gland, liver, epithelium
	Active TSS	Liver	Tissue	Endocrine gland, exocrine gland, liver
	Active TSS	Right lobe of liver	Tissue	Endocrine gland, exocrine gland, liver
rs4794067	Bivalent enhancer	Hepatocyte	Primary cell	Endocrine gland, exocrine gland, liver, epithelium
	Bivalent enhancer	HuH-7.5	Cell line	Endocrine gland, exocrine gland, liver, epithelium
	Bivalent enhancer	HuH-7	Cell line	Endocrine gland, exocrine gland, liver, epithelium
	Bivalent enhancer	Hepatic stellate cell	Primary cell	Endocrine gland, exocrine gland, liver, connective tissue
	Repressed PolyComb	Hepatocyte	In vitro differentiated cells	Endocrine gland, exocrine gland, liver, epithelium
	Repressed PolyComb	HepG2	Cell line	Endocrine gland, exocrine gland, liver, epithelium
	Repressed PolyComb	Right lobe of liver	Tissue	Endocrine gland, exocrine gland, liver
	Repressed PolyComb	Liver	Tissue	Endocrine gland, exocrine gland, liver
	Repressed PolyComb	Liver	Tissue	Endocrine gland, exocrine gland, liver
rs11119982	Repressed PolyComb	Liver	Tissue	Endocrine gland, exocrine gland, liver

*TSS* transcription start site.

**Table 9:** Effect of SNPs on the binding of miRNA (gain or loss).

SNP ID	miRNA (loss)	miRNA (gain)
rs225014 [T/C]	hsa-miR-4712-5p*, hsa-miR-770-5p*	hsa-miR-298*, hsa-miR-148a-3p*, hsa-miR-148b-3p*, hsa-miR-152-3p*, hsa-miR-130a-3p, hsa-miR-130b-3p, hsa-miR-301a-3p, hsa-miR-301b-3p, hsa-miR-3666, hsa-miR-4295, hsa-miR-454-3p, hsa-miR-143-5p, hsa-miR-3944-5p, hsa-miR-1273 h-3p, hsa-miR-3678-3p
rs10865710 [C/G]	hsa-miR-5581-3p*, hsa-miR-8060*, hsa-miR-4709-3p	hsa-miR-520e-5p, hsa-miR-6884-3p
rs532446 [T/C]	hsa-miR-1343-3p, hsa-miR-4299*, hsa-miR-548q, hsa-miR-6783-3p, hsa-miR-7855-5p, hsa-miR-7978	hsa-miR-1272*, hsa-miR-1322, hsa-miR-3682-3p*, hsa-miR-4502*
rs4794067 [T/C]	hsa-miR-1224-3p, hsa-miR-5591-3p, hsa-miR-6512-5p	hsa-miR-4258*, hsa-miR-7108-3p*
rs12031994 [T/C]	hsa-miR-4528*	hsa-miR-1912-5p*, hsa-miR-3618*, hsa-miR-4289*, hsa-miR-934*
rs11119982 [C/T]	hsa-miR-181a-3p*, hsa-miR-4444*, hsa-miR-574-5p*, hsa-miR-3659*	hsa-miR-6513-3p*, hsa-miR-1297*, hsa-miR-26a-5p*, hsa-miR-26b-5p*, hsa-miR-4465*, hsa-miR-595*, hsa-miR-6884-3p

*p values < 0.05; *p* values < 0.01 are marked in bold.

## Discussion

It is well-established that multiple risk factors contribute to cirrhosis and HCC development in CHB patients^16^. Apart from the well-known risk factors such as older age, male gender, chronic active hepatitis, higher ALT levels, or history of decompensation, accumulation of genetic alteration during progression from health, through fibrosis to HCC are now considered of great importance^17^. In this study, we have focused on genetic polymorphism within transcription factor binding sites which are recently suggested as important players in downstream gene expression and phenotypic variations predisposing to different disease development^18^. We have performed an extensive literature review for candidate SNPs located at TFBSs identified by GWAS contributing to complex disease risk. Afterward, we limited the number of loci to those which had a potential impact on TF regulation associated with hepatitis B and/or liver disease progression. As a result, our study demonstrated that rs225014 (*DIO2*), rs532446 (*GADD45A*), rs12031994 (*AKT3*), rs11119982 (*ATF3*), rs10865710 (*PPARG*) might contribute to the increased risk of liver disease progression in chronic hepatitis b carriers. Other parameters including metabolic markers, such as body mass index (BMI), diabetes, and triglyceride levels were not significant in our study. Additionally, no literature data regarding the possible role of investigated SNPs on these variabilities were found.

The strongest prognostic value was found for rs225014 (*DIO2*) and rs532446 (*GADD45A*), which were correlated with liver tissue scaring, as well as with elevated ALT. The CC genotype of *DIO2* rs225014 or C allele occurred more frequently in patients with higher ALT levels, and with more advanced liver fibrosis. Consequently, the C allele had a risk effect for liver disease progression as it was more common in cirrhotic (56%) and HCC (92%) patients. In the same manner, the C allele at rs532446 of *GADD45A* was more common in CHB carriers with both raised ALT concentrations and liver cirrhosis. On the other hand, the TT genotype at both rs225014 (*DIO2*) and rs532446 (*GADD45A*) had a protective effect on liver scarring progression. Additionally, the genotype distribution differed significantly for rs225014 (*DIO2*) between groups of patients affected by different stages of HBV-related liver diseases, and between the cirrhosis and fibrosis group for rs532446 (*GADD45A*). Furthermore, functional mechanisms analysis of these SNPs using computational approaches demonstrated their influence on miRNA binding, target gene expression levels, and different protein binding. To the best of our knowledge, this is the first report presenting an association between polymorphisms of the above genes and the severity of liver disease in CHB patients.

Rs225014 (*DIO2*), also known as Thr92Ala, is involved in thyroid hormone (TH) metabolism and its regulation^19^. This polymorphism was demonstrated to have an impact on TH levels and therefore may influence on a variety of clinical aspects as well as the quality of life or cognition. *DIO2* SNP rs205014 has been so far associated with symptomatic osteoarthritis^20,21^, type 2 diabetes mellitus^22^, atherosclerosis^23^, and bone mineral density^24^ demonstrating the C allele as a risk factor. On the other hand, inversely to our results, the C allele at rs225014 was protective in response to lung injury^25^. Although *DIO2* is not typically expressed in the liver, it has been shown that the lack of the neonatal *DIO2* in mice hepatocytes leads to hepatic epigenetic reprogramming that can alter different liver functions modifying susceptibility to alcohol or diet-induced hepatic steatosis, hypertriglyceridemia, and obesity^26,27^. This may be explained by the fact that the liver is susceptible to the dynamic of THs, which participate in hepatic homeostasis. As the liver is one of the main target tissues of TH, any disruption of TH signals is closely associated with multiple liver-related diseases^28–30^. Moreover, the rs225014 *DIO2*-C allele creates unique TFBS for the NK3 homeobox 2** (**NKX3-2) TF which are eliminated by the T-allele^20,31^. Because homeobox genes are known players in the regulation of HCC tumorigenesis, the elimination of the NKX3-2 binding site by the T-allele the elimination of the NKX3-2 binding site by the T-allele may in part be associated with the protection against liver disease progression. Furthermore, NKX3-2 (also known as BAPX1) has already been demonstrated as a poor prognostic factor for gastric cancer in vivo^32^. Furthermore, the *BAPX1* gene was also reported to be up-regulated in breast and prostate cancers at the mRNA level^33^.

*GADD45A*, TP53-regulated and DNA-damage responsive protein, plays a leading role in human tumorigenesis. Although the exact mechanism remains uncertain, the expression patterns of *GADD45A* vary in different carcinomas^34^. The decreased expression has been observed in patients who suffer from non-small cell lung ^35^ and prostate^36^ cancers. Also *GADD45A* mRNA level was down-regulated in most HCC patients in comparison to adjacent nonneoplastic tissue^37^. GADD45A has been also shown to exert a protective effect against hepatic fibrosis in mice^38^. In contrast, higher *GADD45A* expression was observed in breast cancer tissues compared with non-neoplastic tissue samples^39^. Furthermore, *GADD45A* expression has been associated with the survival of patients with patient esophageal cancer with reduced expression of GADD45A as a poor prognostic factor^40^. Even though the *GADD45A* gene is highly conserved in mammals, point mutations in exon 4 have been found in patients with pancreatic cancer, and *GADD45A* expression combined with p53 status correlated with patients’ survival^41^. Also, rs681673 and rs607375 polymorphisms have been recently found to be associated with breast cancer risk^34^, and *GADD45A* promoter SNP (rs581000) with reduced susceptibility to acute liver injury^42^. Several other studies have found correlations between *GADD45A* polymorphism and ovarian cancer^43^ and rheumatoid arthritis^44^. In our study, we have analyzed located in the p53 binding region an intronic rs532446 (T3812C), which has been previously reported to possess a functional role in acute lung injury^42^. We have found that the minor allele T at rs532446 is associated with decreased susceptibility to liver cirrhosis development. As the SNP is located in the p53 binding region, it may affect the regulatory activity of p53, which is important for both liver homeostasis and dysfunction. On the one hand, p53 regulates cell cycle checkpoints to protect from transformation. On the other hand, it induces apoptosis of damaged cells and activates liver stem/progenitor cells leading to functional recovery of the organ^45^. Therefore it is quite surprising that the minor variant at rs532446 makes a beneficial effect on a patient. However, despite the previously proposed algorithm predicting the affinity of tumor suppressor p53 for binding sites in DNA, response elements containing equal numbers of mismatches still show different affinities for p53. It is probably caused by higher mutation tolerance in an ​unusually long DNA-binding site within which only 20% of nucleotides remain unchanged^46–48^. Moreover, other mechanisms including minor groove shape recognition^49^ and chromatin status^50^ should also be considered when explaining differences in the binding of p53^48^.

In the current study, we also observed an association between enhancer polymorphism rs10865710 in the *PPARG* gene and liver fibrosis progression. Although a C → G substitution at this site does not cause an amino acid change, rs10865710 was proposed as a risk factor for a variety of diseases^51^, such as asthma^52^, systemic sclerosis^53^, obesity^54^, as well as a non-alcoholic fatty liver disease^55^. Moreover, Lu et al*.*^51^ have recently demonstrated that carriers with rs10865710 CG/GG genotypes express lower levels of *PPARG* in comparison to individuals with CC genotype, which may be associated with the downregulation of *PPARG* expression. Additionally, hepatic *PPARG* expression has been noted to promote liver steatosis^56^, and inhibition of *PPARG* has been shown to suppress steatosis-associated liver cancer in mice^57^. Associated with lower *PPARG* level rs10865710 minor allele was more common in patients with low fibrosis scores in our study. Of the six unique TFBS generated by the G allele, MEIS1 has been already shown to play a role in cardiovascular regeneration^58^. Because inhibitory effects of MEIS1 on tumorigenesis in renal clear cell carcinoma^59^, non-small-cell lung cancer cells ^60^, or prostate cancer^61^ have been reported, we suppose that the creation of the MEIS1 TFBS with the minor G allele may in part be responsible for the association of this SNP with liver fibrosis risk. In the same manner, associated with cirrhosis risk rs11119982 (*ATF3*) C allele creates one unique TFBS for the helicase-like transcription factor (HLTF) which is involved with altering chromatin structure. On the other hand, the minor T allele at this site is located in the binding site of five TFs that regulate transcription, and control hematopoietic progenitor cell control, cellular transcription, and repression.

This cross-sectional study has some limitations. Although we have analyzed ALT levels within groups with different liver damage scores, these measurements were performed at the time of liver assessment and we have no information regarding the further progression of the liver. Given that ALT is tend to fluctuate, people with early stages of liver cirrhosis can have normal liver function tests. Secondly, our study was performed on Caucasian subjects only. Therefore, similar studies on other geographic regions with different genetic populations should be done.

This study showed that rs225014 (*DIO2*), rs532446 (*GADD45A*), rs12031994 (*AKT3*), rs11119982 (*ATF3*), rs10865710 (*PPARG*) are associated with the increased risk of liver disease progression in chronic hepatitis b carriers. The presence of the C allele at both *DIO2* rs225014 and *GADD45A* rs532446 was independently associated with liver tissue scarring. Moreover, the occurrence of HCC in the study group was more common in individuals carrying the rs225014 CC genotype, and the rs532446 together with rs11119982 were associated with liver cirrhosis development.

## Materials and methods

### Patients

This study included 284 patients with confirmed CHB infection (HBsAg positive for more than 6 months) from the ANRS CO22 HEPATHER cohort (ClinicalTrials.gov registry number: NCT01953458). All the subjects were of Caucasian ethnicity and had no other concomitant liver etiologies (viral coinfection, autoimmune or metabolic). Patients were excluded if they were currently treated or had undergone antiviral treatment within 6 months before the initiation of the study. Serum samples were collected before liver fibrosis assessment, and underwent the standard procedure in the local clinical center laboratory, including hepatitis serologic variables (HBsAg, HBsAb, HBeAg, HBeAb, HBcAb, HBV DNA levels). Fibrosis scores were assessed by non-invasive transient elastography by using FibroScan (Echosens, Paris, France), and the METAVIR scoring system^62^ was used for patient classification. Patients were subdivided as follows: no fibrosis (no scarring, stage 0), mild fibrosis (fibrosis stage I), liver fibrosis (fibrosis stages II-III), and cirrhosis (fibrosis stage IV; confirmed by two experienced pathologists). The procedures employed followed the ethical standards of the 1975 Declaration of Helsinki revised in 2013. The study protocol was approved by the Local Independent Bioethics Committee and the ANRS CO22 HEPATHER scientific committee. We have received the agreement to use the HEPATHER cohort in the frame of the INFECT-ERA project, and access to samples was paid for by the University of Gdansk. All enrolled subjects signed a free and informed consent form for participation in the study.

### SNP genotyping

DNA was extracted from the whole blood samples (200 uL) using the MagNa Pure LC DNA Isolation System, and according to the standard manufacturer protocol for MagNA Pure Compact Nucleic Acid Isolation Kit I (Roche, Mannheim, Germany). The genotypes were determined by mass spectrometry method with the use of an iPLEX Pro chemistry for single base extension reaction according to the protocol (Agena Bioscience, San Diego, CA, USA). For each SNP, one primer pair and a single extension primer sequence were designed using the Mass Assay Designer software package (v.4.0). All primer sequences are listed in Table S6. Out of the nine SNPs included in the study, only one was localized within the transcription factor (*TBX21*, rs4794067). The remaining SNPs identified at the transcription factor binding site (TFBS) include *DIO2* (rs225017, rs225014), *PPARG* (rs10865710, rs2016520), *ATF3 (*rs11119982), *AKT3* (rs12031994), and *GADD45A* (rs532446, rs37834688).

41 µL of ultrapure water was used to dilute the final extension product following the transfer into Chip Prep Module (Agena Bioscience, San Diego, CA, USA) for automated sample handling including desalting and dispensing samples onto the SpectroChip Array (Agena Bioscience, San Diego, CA, USA). Mass spectra were acquired with a MassARRAY^®^ Analyzer 4 mass spectrometer and analyzed with MassARRAY^®^ Typer 4.0 software. All procedures were performed according to the company’s recommendations.

### Statistical analysis

Statistical analyses were performed using STATISTICA software version 13.3 (StatSoft, Tulsa OK, USA). The Hardy–Weinberg equilibrium of analyzed SNPs was conducted by the MIDAS software. Chi-squared or Fisher’s exact test was used to analyze the relationship between categorical vs. categorical variables. Logistic regression analysis was used to evaluate the contribution of genetic and nongenetic factors under the dominant, recessive, and additive models. A backward stepwise regression approach was applied when building multivariate models. All of the *p*-values presented were two-sided and only *p* < 0.05 was considered significant.

### Bioinformatics analysis of statistically significant SNPs

Four software were used to analyze the effect of rs225014 on DIO2 protein. SIFT web server (https://sift.bii.a-star.edu.sg/www/SIFT_seq_submit2.html) was used to predict SNP impact on protein function based on sequence homology and the physical properties of amino acids. A score below or equal to 0.05 in a range between 0 and 1 conferred the deleterious effect of SNP on protein function. MUpro (http://mupro.proteomics.ics.uci.edu/) and I-mutant 3.0 (http://gpcr2.biocomp.unibo.it/cgi/predictors/I-Mutant3.0/I-Mutant3.0.cgi) web tools were used to determine whether the Thr92Ala amino acid substitution affects DIO2 protein’s stability. Structural and functional effects of rs225014 were analyzed by the HOPE (Have (y) Our Protein Explained) (https://www3.cmbi.umcn.nl/hope/) server. MetaRNN pathogenicity prediction score was used (range 0–1), which when higher shows higher pathogenicity.

Investigation of any potential harmful effect of non-coding SNPs was performed at Regulome DB v2.1 (https://beta.regulomedb.org/regulome-search//), which gives a ranking based on DNA binding, provides Chip data, chromatin states, and motifs. The RegulomeDB probability score is ranging from 0 to 1, with 1 being the most likely to be a regulatory variant. Furthermore, to predict the target gain/loss effect of SNPs in miRNA seed regions, miRNASNP was employed (miRNASNP-v3 (hust.edu.cn)).

## Supplementary Information

Supplementary Tables.

## Data Availability

The datasets generated and analyzed during the current study are available in the BioStudies database, S-BSST1042.
