# Supplementary material for: Polymorphisms within DIO2 and GADD45A genes increase the risk of liver disease progression in chronic hepatitis b carriers
Source: Sci Rep. 2023 Apr 14;13:6124. doi: 10.1038/s41598-023-32753-8 (PMC10104815; doi:10.1038/s41598-023-32753-8)
Supplement: Supplementary file 1 — Supplementary Tables. [file 41598_2023_32753_MOESM1_ESM.pdf]

Table S1. Gene polymorphisms associated with advanced liver fibrosis.

Genotype	MAF	Odds ratio	95% CI	*P value*
***DIO2* rs225014 [T/C]**				
CC, CT vs TT	C=0.46	0.55	0.34-0.89	***0.015***
***DIO2* rs225017 [T/A]**				
TT,TA vs. AA	A=0.31	0.57	0.34-0.94	***0.026***
***PPARG* rs10865710 [G/C]**				
CC,CG vs GG	G=0.26	1.94	1.21-3.14	***0.006***

Sex and age adjusted logistic regression analysis. MAF – minor allele frequency.

Table S2. Chip-seq data obtained for rs532446 by Regulome DB.

Peak location	Biosample	Targets	Organ	Value
chr1:67686165..67687054	transverse colon	POLR2A	colon, large intestine, intestine	236.70162
chr1:67686539..67686839	HepG2	NR2F1	endocrine gland, exocrine gland, liver, epithelium	19.30739
chr1:67686534..67687034	HepG2	RCOR2	endocrine gland, exocrine gland, liver, epithelium	24.55665
chr1:67686541..67687061	HepG2	MXD3	endocrine gland, exocrine gland, liver, epithelium	19.33018
chr1:67686679..67687015	HEK293	TSHZ1	kidney, epithelium	97.57680
chr1:67686597..67686967	HepG2	TFAP4	endocrine gland, exocrine gland, liver, epithelium	30.57992
chr1:67686284..67686964	K562	ZNF175	bodily fluid, blood	49.40842
chr1:67686646..67687090	A549	FOSL2	lung	45.70996
chr1:67686271..67686841	mesothelial cell of epicardium	CTCF	heart, pericardium, epithelium	11.00976
chr1:67686502..67686946	upper lobe of left lung	POLR2A	lung	31.37125
chr1:67686486..67686966	GM12878	HDGF	bodily fluid, blood	66.80010
chr1:67686516..67687036	HepG2	TARDBP	endocrine gland, exocrine gland, liver, epithelium	33.91263

chr1:67686576..67686912	excitatory neuron	ID3		15.86863
chr1:67686289..67686849	K562	CXXC5	bodily fluid, blood	95.71556
chr1:67686648..67686968	K562	PHF21A	bodily fluid, blood	51.63659
chr1:67686573..67686857	vagina	POLR2A	vagina	26.85197
chr1:67686749..67687059	HCT116	RAD21	colon, large intestine, epithelium, intestine	12.98610
chr1:67686730..67686999	A549	JUN	lung	81.67566
chr1:67686680..67687380	A549	JUN	lung	27.06940
chr1:67686599..67687099	A549	JUNB	lung	40.72242
chr1:67686544..67687188	A549	BCL3	lung	56.64945
chr1:67686624..67686878	GM12878	RELB	bodily fluid, blood	86.40458
chr1:67686581..67687001	HepG2	ETV4	endocrine gland, exocrine gland, liver, epithelium	49.48126
chr1:67684876..67686840	Spleen	POLR2A	spleen, immune organ	166.35341
chr1:67684876..67686840	Spleen	POLR2A	spleen, immune organ	3949.13931
chr1:67686652..67686966	A549	JUN	lung	67.65756
chr1:67686291..67687011	A549	JUN	lung	67.24156
chr1:67686330..67687003	gastrocnemius medialis	POLR2A	musculature of body, limb	371.53276
chr1:67686562..67687086	A549	JUNB	lung	57.85954
chr1:67686630..67687046	A549	FOSL2	lung	27.04981
chr1:67686372..67687236	K562	ZBTB11	bodily fluid, blood	67.95985
chr1:67685614..67687036	HEK293	PATZ1	kidney, epithelium	97.64855
chr1:67686501..67686881	HepG2	KDM6A	endocrine gland, exocrine gland, liver, epithelium	26.13204
chr1:67686301..67686891	HepG2	ZNF709	endocrine gland, exocrine gland, liver, epithelium	143.66984
chr1:67686621..67687071	HepG2	ZNF121	endocrine gland, exocrine gland, liver, epithelium	19.22967
chr1:67686361..67686811	HepG2	ZNF121	endocrine gland, exocrine gland, liver, epithelium	52.38463

chr1:67686640..67686996	HEK293	OVOL3	kidney, epithelium	62.43639
chr1:67686718..67687014	K562	GATA1	bodily fluid, blood	17.33054
chr1:67686113..67686783	HepG2	SNAPC4	endocrine gland, exocrine gland, liver, epithelium	35.88217
chr1:67686373..67686993	HepG2	ZNF598	endocrine gland, exocrine gland, liver, epithelium	63.24681
chr1:67686498..67686769	Peyer's patch	POLR2A	intestine, lymphoid tissue	203.54043
chr1:67686586..67686946	HEK293	ZNF146	kidney, epithelium	55.32253
chr1:67686444..67687000	HepG2	EGR1	endocrine gland, exocrine gland, liver, epithelium	21.95730
chr1:67686485..67686995	K562	CEBPG	bodily fluid, blood	113.00191
chr1:67686297..67686761	K562	NONO	bodily fluid, blood	19.00862
chr1:67684917..67687504	K562	POLR2A	bodily fluid, blood	343.09513
chr1:67684917..67687504	K562	POLR2A	bodily fluid, blood	119.28240
chr1:67686734..67686899	K562	TRIM24	bodily fluid, blood	131.72188
chr1:67686636..67687106	HepG2	ZFHX3	endocrine gland, exocrine gland, liver, epithelium	33.15878
chr1:67686537..67687173	A549	BCL3	lung	29.81089
chr1:67686629..67687069	A549	FOSL2	lung	36.82996
chr1:67686691..67686979	K562	MAFG	bodily fluid, blood	315.91348
chr1:67686616..67687032	K562	TCF12	bodily fluid, blood	446.51885
chr1:67686608..67686992	HEK293	ATF2	kidney, epithelium	62.80102
chr1:67686124..67686828	22Rv1	CTCF	prostate gland	75.81501
chr1:67686261..67686901	HepG2	SAFB2	endocrine gland, exocrine gland, liver, epithelium	33.03112
chr1:67686465..67687101	HepG2	GMEB1	endocrine gland, exocrine gland, liver, epithelium	32.69632
chr1:67686523..67687093	HepG2	ZNF574	endocrine gland, exocrine gland, liver, epithelium	39.07901
chr1:67686615..67687191	K562	GATA2	bodily fluid, blood	54.58057
chr1:67686401..67686805	WTC11	TEAD1	skin of body, connective tissue	26.28470

chr1:67686685..67686990	A549	JUN	lung	40.99073
chr1:67686385..67686821	HepG2	TAF1	endocrine gland, exocrine gland, liver, epithelium	45.25706
chr1:67686718..67686974	A549	USF1	lung	20.47963
chr1:67686695..67686908	HEK293	ZBTB21	kidney, epithelium	149.62044
chr1:67686447..67687096	HEK293	ZXDB	kidney, epithelium	269.46024
chr1:67686486..67686926	A673	CTCF	musculature of body	6.59824
chr1:67686366..67686896	A549	SIN3A	lung	112.00955
chr1:67686499..67686809	A549	POLR2A	lung	24.83275
chr1:67686322..67686882	K562	NRF1	bodily fluid, blood	94.27060
chr1:67686678..67686962	HeLa-S3	NFE2L2	uterus, epithelium	37.13291
chr1:67686660..67687014	K562	HMBOX1	bodily fluid, blood	117.08667
chr1:67686738..67686954	SK-N-SH	RAD21	brain	51.96689
chr1:67686734..67686981	K562	EP300	bodily fluid, blood	194.95079
chr1:67686473..67686937	K562	HMGN3	bodily fluid, blood	42.17926
chr1:67686673..67687137	K562	HMGN3	bodily fluid, blood	11.66317
chr1:67686697..67686993	endothelial cell of umbilical vein	JUN	blood vessel, vein, placenta, extraembryonic component, vasculature, epithelium	73.65379
chr1:67686683..67687003	HEK293	ZNF596	kidney, epithelium	90.83358
chr1:67686235..67686759	WTC11	FOXP1	skin of body, connective tissue	52.73508
chr1:67686542..67687002	Ishikawa	MAX	uterus	32.33595
chr1:67686604..67687034	PFSK-1	TAF1	connective tissue, brain	40.15480
chr1:67686630..67687010	SK-N-SH	ZBTB33	brain	38.42263
chr1:67686546..67687090	K562	TFAP4	bodily fluid, blood	60.37271
chr1:67686338..67686882	K562	TFAP4	bodily fluid, blood	89.25981
chr1:67686658..67687054	HEK293	ZNF580	kidney, epithelium	62.17032

chr1:67686724..67687004	K562	MAFK	bodily fluid, blood	102.65206
chr1:67684816..67686979	HepG2	ZNF687	endocrine gland, exocrine gland, liver, epithelium	523.90232
chr1:67684816..67686979	HepG2	ZNF687	endocrine gland, exocrine gland, liver, epithelium	245.67412
chr1:67686231..67686835	HepG2	MTA1	endocrine gland, exocrine gland, liver, epithelium	37.00757
chr1:67686728..67687038	SK-N-SH	USF1	brain	23.86331
chr1:67686299..67687148	HEK293	ZNF629	kidney, epithelium	496.04701
chr1:67686674..67687070	HCT116	FOSL1	colon, large intestine, epithelium, intestine	107.89130
chr1:67686712..67687012	K562	GATA2	bodily fluid, blood	57.47556
chr1:67686537..67687017	A549	SMC3	lung	37.01892
chr1:67686598..67687068	GM12878	TARDBP	bodily fluid, blood	60.48203
chr1:67686427..67686957	HepG2	ZEB1	endocrine gland, exocrine gland, liver, epithelium	32.54429
chr1:67686395..67687015	HepG2	TBX2	endocrine gland, exocrine gland, liver, epithelium	79.02526
chr1:67686621..67687037	HEK293	ZNF558	kidney, epithelium	80.93666
chr1:67686672..67687036	HEK293	ZSCAN23	kidney, epithelium	88.20695
chr1:67686125..67687142	HEK293	ZBTB17	kidney, epithelium	481.29870
chr1:67686687..67687057	HepG2	TEAD3	endocrine gland, exocrine gland, liver, epithelium	20.45732
chr1:67686645..67687069	MCF-7	FOS	mammary gland, exocrine gland, epithelium	559.44591
chr1:67686576..67686912	HepG2	NFIL3	endocrine gland, exocrine gland, liver, epithelium	41.46972
chr1:67686629..67686970	GM12878	IKZF2	bodily fluid, blood	350.21814
chr1:67686548..67686944	HepG2	TBX3	endocrine gland, exocrine gland, liver, epithelium	63.53544
chr1:67686545..67687044	K562	HDAC1	bodily fluid, blood	13.15247
chr1:67686683..67686898	HepG2	ETV5	endocrine gland, exocrine gland, liver, epithelium	97.64477
chr1:67684943..67687064	HepG2	POLR2G	endocrine gland, exocrine gland, liver, epithelium	440.74301
chr1:67684943..67687064	HepG2	POLR2G	endocrine gland, exocrine gland, liver, epithelium	98.16179

chr1:67686650..67686974	stomach	POLR2A	stomach	56.83898
chr1:67686617..67687127	K562	TEAD1	bodily fluid, blood	81.18428
chr1:67686144..67686764	K562	ARID4B	bodily fluid, blood	35.47877
chr1:67686525..67687265	K562	ZNF589	bodily fluid, blood	42.66031
chr1:67686565..67686943	K562	POLR2A	bodily fluid, blood	85.53822
chr1:67686429..67686973	middle frontal area 46	CTCF	brain	21.30717
chr1:67686684..67687020	IMR-90	RCOR1	lung, connective tissue	30.74973
chr1:67686639..67687045	K562	ZEB2	bodily fluid, blood	225.78874
chr1:67686624..67687040	MCF-7	CTBP1	mammary gland, exocrine gland, epithelium	47.90949
chr1:67686516..67687012	HepG2	KDM3A	endocrine gland, exocrine gland, liver, epithelium	16.77688
chr1:67686618..67687222	K562	ZNF766	bodily fluid, blood	38.30919
chr1:67686560..67687227	K562	FOSL1	bodily fluid, blood	195.35405
chr1:67686615..67686939	HepG2	EP300	endocrine gland, exocrine gland, liver, epithelium	49.87516
chr1:67686743..67687079	K562	HDAC1	bodily fluid, blood	66.20997
chr1:67686402..67686892	GM23338	POLR2A	skin of body	15.13110
chr1:67686512..67687102	K562	SKIL	bodily fluid, blood	122.08546
chr1:67686661..67686971	MCF-7	MYC	mammary gland, exocrine gland, epithelium	31.94545
chr1:67686717..67687201	K562	ARNT	bodily fluid, blood	32.64890
chr1:67686479..67687095	A549	BCL3	lung	74.99915
chr1:67686681..67687297	A549	BCL3	lung	35.19264
chr1:67686732..67686962	GM12878	BATF	bodily fluid, blood	52.80787
chr1:67686731..67687022	K562	NFIC	bodily fluid, blood	273.74206
chr1:67686636..67687036	K562	MNT	bodily fluid, blood	220.24569
chr1:67686474..67686844	K562	SMAD4	bodily fluid, blood	59.10707

chr1:67686547..67687125	K562	ARID1B	bodily fluid, blood	1361.20058
chr1:67686224..67686768	middle frontal area 46	CTCF	brain	31.59188
chr1:67686707..67687028	K562	TAL1	bodily fluid, blood	350.54804
chr1:67686559..67686983	sigmoid colon	POLR2A	colon, large intestine, intestine	42.51319
chr1:67686705..67686809	HCT116	CEBPB	colon, large intestine, epithelium, intestine	152.74612
chr1:67686293..67687203	K562	NR2C2	bodily fluid, blood	81.44855
chr1:67685895..67686805	K562	NR2C2	bodily fluid, blood	77.17526
chr1:67686510..67687420	K562	NR2C2	bodily fluid, blood	36.30621
chr1:67686574..67687154	K562	SMARCA4	bodily fluid, blood	22.46218
chr1:67686515..67686892	HEK293	ZBTB48	kidney, epithelium	143.49591
chr1:67686447..67687083	HepG2	ZNF605	endocrine gland, exocrine gland, liver, epithelium	71.43815
chr1:67686456..67686856	K562	E2F5	bodily fluid, blood	44.22842
chr1:67686521..67686816	MCF 10A	POLR2A	mammary gland, exocrine gland, epithelium	58.10463
chr1:67686731..67686996	K562	ZEB2	bodily fluid, blood	166.99166
chr1:67686727..67687291	K562	SIN3A	bodily fluid, blood	21.00917
chr1:67686685..67687012	K562	SOX6	bodily fluid, blood	270.09088
chr1:67686686..67687046	SK-N-SH	CHD2	brain	42.85920
chr1:67686593..67686937	GM18951	RELA	bodily fluid, blood	26.04506
chr1:67686563..67687143	MCF-7	FOSL2	mammary gland, exocrine gland, epithelium	63.41588
chr1:67686316..67686900	K562	TFDP1	bodily fluid, blood	31.18230
chr1:67686707..67687107	SK-N-SH	TEAD4	brain	83.54654
chr1:67686708..67686980	MCF 10A	FOS	mammary gland, exocrine gland, epithelium	193.01058
chr1:67686545..67687016	K562	POLR2A	bodily fluid, blood	71.65184
chr1:67686475..67687011	GM12878	GATAD2B	bodily fluid, blood	39.17443

chr1:67686703..67687299	K562	CHD4	bodily fluid, blood	8.97922
chr1:67686621..67686881	HepG2	HNF4A	endocrine gland, exocrine gland, liver, epithelium	29.64538
chr1:67686482..67686942	K562	POLR2A	bodily fluid, blood	142.12405
chr1:67686460..67687238	K562	MLLT1	bodily fluid, blood	149.73938
chr1:67686718..67686889	K562	ETV1	bodily fluid, blood	312.91830
chr1:67686679..67686989	K562	BACH1	bodily fluid, blood	54.63217
chr1:67686645..67687049	A549	FOSL2	lung	57.86701
chr1:67686583..67687013	K562	CBX3	bodily fluid, blood	39.95291
chr1:67686547..67687143	GM12878	DPF2	bodily fluid, blood	75.08296
chr1:67686422..67687102	K562	E2F5	bodily fluid, blood	32.48296
chr1:67686555..67686835	esophagus squamous	POLR2A	epithelium, esophagus	39.61583
chr1:67686648..67687038	K562	ATF2	bodily fluid, blood	64.82129
chr1:67686240..67686933	K562	SMAD5	bodily fluid, blood	105.64237
chr1:67686550..67686783	breast epithelium	POLR2A	breast, epithelium	77.47435
chr1:67686731..67687037	MCF 10A	STAT3	mammary gland, exocrine gland, epithelium	125.26798
chr1:67686525..67686801	HepG2	ELF1	endocrine gland, exocrine gland, liver, epithelium	16.10416
chr1:67686683..67686973	SK-N-SH	JUND	brain	106.47775
chr1:67686725..67686985	HeLa-S3	SMC3	uterus, epithelium	38.25500
chr1:67686586..67687100	K562	SMARCE1	bodily fluid, blood	336.81764
chr1:67686526..67686846	HepG2	GTF2F1	endocrine gland, exocrine gland, liver, epithelium	19.71057
chr1:67686609..67687083	K562	EP400	bodily fluid, blood	385.14089
chr1:67686538..67687128	GM12878	NFIC	bodily fluid, blood	36.22135
chr1:67686634..67687104	Ishikawa	TCF12	uterus	42.60353
chr1:67686444..67687014	GM12878	NFATC3	bodily fluid, blood	70.58631

chr1:67686644..67687214	GM12878	NFATC3	bodily fluid, blood	21.24309
chr1:67685072..67686828	HepG2	AGO2	endocrine gland, exocrine gland, liver, epithelium	105.55437
chr1:67685072..67686828	HepG2	AGO2	endocrine gland, exocrine gland, liver, epithelium	87.01426
chr1:67686712..67686837	HepG2	SOX13	endocrine gland, exocrine gland, liver, epithelium	66.31978
chr1:67686195..67686979	H1	ASH2L	embryo	29.94186
chr1:67686666..67686872	HepG2	DMAP1	endocrine gland, exocrine gland, liver, epithelium	46.58672
chr1:67686514..67686958	heart left ventricle	POLR2A	heart	73.77526
chr1:67686687..67687041	K562	NCOR1	bodily fluid, blood	587.37309
chr1:67686264..67686800	A549	BCL3	lung	19.96473
chr1:67686702..67687022	K562	PYGO2	bodily fluid, blood	52.22308
chr1:67686354..67686844	HepG2	HNRNPL	endocrine gland, exocrine gland, liver, epithelium	8.69396
chr1:67686698..67687078	K562	DACH1	bodily fluid, blood	146.59777
chr1:67686316..67686766	K562	TARDBP	bodily fluid, blood	56.07721
chr1:67686417..67686967	K562	GABPB1	bodily fluid, blood	59.48562
chr1:67686677..67686975	A549	JUN	lung	92.36012
chr1:67686464..67687000	K562	MAX	bodily fluid, blood	65.44664
chr1:67686528..67686918	gastroesophageal sphincter	POLR2A	musculature of body, stomach	73.39003
chr1:67686391..67686947	K562	NRF1	bodily fluid, blood	29.30606
chr1:67686649..67687031	K562	EMSY	bodily fluid, blood	497.92240
chr1:67686584..67687042	K562	NCOR1	bodily fluid, blood	354.16848
chr1:67686285..67686801	HepG2	ZNF33B	endocrine gland, exocrine gland, liver, epithelium	45.58382
chr1:67686274..67686849	HEK293	ZBTB20	kidney, epithelium	24.19539
chr1:67686607..67687006	transverse colon	POLR2A	colon, large intestine, intestine	178.69087
chr1:67686487..67687017	MCF-7	SIN3A	mammary gland, exocrine gland, epithelium	44.67888

chr1:67686609..67687065	A549	EP300	lung	23.25762
chr1:67684889..67687202	K562	POLR2G	bodily fluid, blood	273.78327
chr1:67684889..67687202	K562	POLR2G	bodily fluid, blood	221.02864
chr1:67686683..67686849	HepG2	RXRB	endocrine gland, exocrine gland, liver, epithelium	65.52470
chr1:67686444..67686900	HepG2	ZNF441	endocrine gland, exocrine gland, liver, epithelium	34.65554
chr1:67684910..67690102	HepG2	RBFOX2	endocrine gland, exocrine gland, liver, epithelium	185.92927
chr1:67684910..67690102	HepG2	RBFOX2	endocrine gland, exocrine gland, liver, epithelium	98.65314
chr1:67686714..67686867	HepG2	TFAP4	endocrine gland, exocrine gland, liver, epithelium	47.04836
chr1:67686625..67686873	HEK293	ZNF692	kidney, epithelium	100.91924
chr1:67686640..67687120	K562	IKZF1	bodily fluid, blood	621.99967
chr1:67685038..67687053	K562	POLR2A	bodily fluid, blood	140.92380
chr1:67685038..67687053	K562	POLR2A	bodily fluid, blood	66.50783
chr1:67686699..67686814	K562	CEBPG	bodily fluid, blood	269.75764
chr1:67686680..67686956	NB4	MAX	bone marrow, bone element	53.59214
chr1:67686748..67686988	K562	JUNB	bodily fluid, blood	114.40519
chr1:67686651..67686994	HEK293	KLF8	kidney, epithelium	174.60956
chr1:67686734..67687016	HEK293	KLF1	kidney, epithelium	162.75840
chr1:67685716..67686960	HEK293	MAZ	kidney, epithelium	115.92245
chr1:67685716..67686960	HEK293	MAZ	kidney, epithelium	30.11700
chr1:67685716..67686960	HEK293	MAZ	kidney, epithelium	182.91123
chr1:67686595..67687131	HepG2	NFIC	endocrine gland, exocrine gland, liver, epithelium	39.08704
chr1:67686642..67686958	NB4	MYC	bone marrow, bone element	44.62205
chr1:67686442..67686946	HEK293	ZNF501	kidney, epithelium	151.69717
chr1:67686560..67687116	HEK293	SP3	kidney, epithelium	74.77248

chr1:67686606..67687126	HEK293	ZNF341	kidney, epithelium	55.27334
chr1:67686666..67686996	HEK293	KLF10	kidney, epithelium	204.34863
chr1:67686490..67687080	liver	YY1	endocrine gland, exocrine gland, liver	64.47635
chr1:67686614..67686954	transverse colon	POLR2A	colon, large intestine, intestine	23.31285
chr1:67686687..67687057	MCF-7	GATA3	mammary gland, exocrine gland, epithelium	63.20045
chr1:67686621..67686985	HepG2	JUND	endocrine gland, exocrine gland, liver, epithelium	31.51530
chr1:67686281..67687008	HEK293	ZNF600	kidney, epithelium	487.06534
chr1:67686620..67687044	HEK293	ZNF34	kidney, epithelium	34.40246
chr1:67686691..67686891	K562	TAF1	bodily fluid, blood	30.80713
chr1:67686564..67687154	K562	NFRKB	bodily fluid, blood	48.00301
chr1:67686386..67686770	K562	UBTF	bodily fluid, blood	22.22018
chr1:67686497..67686785	HepG2	POLR2A	endocrine gland, exocrine gland, liver, epithelium	58.55229
chr1:67686679..67687029	K562	BCLAF1	bodily fluid, blood	13.62571
chr1:67686737..67687087	K562	IRF1	bodily fluid, blood	48.44473
chr1:67686555..67686965	K562	REST	bodily fluid, blood	67.26076
chr1:67686448..67686998	K562	CREB3L1	bodily fluid, blood	84.29174
chr1:67686534..67687127	K562	ATF3	bodily fluid, blood	750.55387
chr1:67686523..67686960	HepG2	ARID4B	endocrine gland, exocrine gland, liver, epithelium	66.23357
chr1:67686583..67686960	HepG2	ARID4B	endocrine gland, exocrine gland, liver, epithelium	27.53475
chr1:67686531..67686961	HepG2	ZNF219	endocrine gland, exocrine gland, liver, epithelium	54.19963
chr1:67686650..67687126	A549	EP300	lung	36.60317
chr1:67686732..67687079	endothelial cell of umbilical vein	GATA2	blood vessel, vein, placenta, extraembryonic component, vasculature, epithelium	259.93505
chr1:67686500..67687280	K562	ZNF395	bodily fluid, blood	59.92385
chr1:67686705..67687485	K562	ZNF395	bodily fluid, blood	15.15631

chr1:67686688..67687012	HeLa-S3	EP300	uterus, epithelium	92.02337
chr1:67686559..67686989	A549	EP300	lung	16.21723
chr1:67686671..67686992	HeLa-S3	TBP	uterus, epithelium	111.63630
chr1:67686317..67686901	K562	GABPB1	bodily fluid, blood	73.95007
chr1:67686187..67686909	vagina	POLR2A	vagina	194.58985
chr1:67686187..67686909	vagina	POLR2A	vagina	29.00997
chr1:67686630..67687080	A549	FOSL2	lung	27.92977
chr1:67686639..67687075	A549	FOSL2	lung	29.60115
chr1:67686738..67687102	K562	PYGO2	bodily fluid, blood	46.31171
chr1:67686572..67687012	K562	CTBP1	bodily fluid, blood	299.98856
chr1:67686235..67686775	LNCAP	CTCF	prostate gland	95.35586
chr1:67686585..67686955	HEK293	ZNF654	kidney, epithelium	34.26137
chr1:67686400..67686824	K562	ZNF24	bodily fluid, blood	39.08626
chr1:67686496..67686912	gastroesophageal sphincter	POLR2A	musculature of body, stomach	29.24586
chr1:67686621..67686897	suprapubic skin	POLR2A	skin of body	74.55235
chr1:67686668..67687092	K562	KLF1	bodily fluid, blood	24.74608
chr1:67686313..67686777	A549	POLR2A	lung	61.53837
chr1:67686727..67687277	GM12878	SKIL	bodily fluid, blood	24.36212
chr1:67686654..67687074	liver	JUND	endocrine gland, exocrine gland, liver	76.39656
chr1:67686016..67686760	K562	ZNF584	bodily fluid, blood	24.78894
chr1:67686703..67687153	HEK293	ZSCAN4	kidney, epithelium	24.66636
chr1:67686535..67687035	K562	KDM1A	bodily fluid, blood	44.30518
chr1:67686667..67686923	HeLa-S3	GABPA	uterus, epithelium	13.34625
chr1:67686599..67687019	K562	NCOA1	bodily fluid, blood	91.58037

chr1:67686574..67687090	A549	SP1	lung	63.06775
chr1:67686340..67686824	H1	TAF1	embryo	20.87501
chr1:67686656..67686799	HepG2	SKI	endocrine gland, exocrine gland, liver, epithelium	95.59885
chr1:67686682..67686992	HEK293	KLF17	kidney, epithelium	169.50317
chr1:67686610..67687100	HEK293	ZFP37	kidney, epithelium	97.77336
chr1:67686686..67687142	K562	NR2F6	bodily fluid, blood	69.38776
chr1:67686641..67686891	HeLa-S3	CEBPB	uterus, epithelium	154.40298
chr1:67686496..67686986	liver	NR2F2	endocrine gland, exocrine gland, liver	38.95050
chr1:67686688..67686892	K562	SPI1	bodily fluid, blood	21.42221
chr1:67686479..67686909	breast epithelium	POLR2A	breast, epithelium	67.87100
chr1:67686683..67687113	breast epithelium	POLR2A	breast, epithelium	12.10223
chr1:67686536..67686896	HEK293	ZSCAN5A	kidney, epithelium	47.19444
chr1:67686618..67687074	K562	LEF1	bodily fluid, blood	120.90024
chr1:67686630..67686900	GM12878	BCL11A	bodily fluid, blood	20.95417
chr1:67686383..67686867	K562	HDGF	bodily fluid, blood	66.26257
chr1:67686562..67686922	HepG2	TBP	endocrine gland, exocrine gland, liver, epithelium	55.69984
chr1:67686720..67687056	HeLa-S3	STAT3	uterus, epithelium	36.17487
chr1:67686741..67686975	SK-N-SH	JUND	brain	159.99778
chr1:67686563..67687003	HEK293	ZNF518A	kidney, epithelium	44.35384
chr1:67686680..67686950	HEK293	ZSCAN30	kidney, epithelium	157.74061
chr1:67686658..67687114	SK-N-SH	FOXM1	brain	37.04265
chr1:67686566..67687010	HepG2	NFYA	endocrine gland, exocrine gland, liver, epithelium	23.07557
chr1:67686499..67686959	HepG2	MED1	endocrine gland, exocrine gland, liver, epithelium	87.24570
chr1:67686391..67687061	HepG2	NR2C2	endocrine gland, exocrine gland, liver, epithelium	42.66341

chr1:67686189..67686869	22Rv1	CTCF	prostate gland	75.12275
chr1:67686451..67686775	uterus	CTCF	uterus	26.32670
chr1:67686345..67687095	HepG2	ZNF761	endocrine gland, exocrine gland, liver, epithelium	38.48613
chr1:67686501..67686981	liver	MAX	endocrine gland, exocrine gland, liver	59.28194
chr1:67686597..67686889	K562	CEBPB	bodily fluid, blood	344.01228
chr1:67686597..67687067	K562	NBN	bodily fluid, blood	78.61435
chr1:67686195..67686805	HepG2	ZNF883	endocrine gland, exocrine gland, liver, epithelium	47.20481
chr1:67686470..67687046	HepG2	NCOR1	endocrine gland, exocrine gland, liver, epithelium	27.20054
chr1:67686561..67687017	A549	CEBPB	lung	40.41217
chr1:67686667..67687023	HEK293	ZNF184	kidney, epithelium	52.03917
chr1:67686663..67687083	HEK293	PRDM1	kidney, epithelium	81.27011
chr1:67686725..67686931	HEK293	ZNF639	kidney, epithelium	125.54699
chr1:67686502..67687042	HepG2	ERF	endocrine gland, exocrine gland, liver, epithelium	51.20151
chr1:67686558..67686898	adrenal gland	POLR2A	endocrine gland, adrenal gland	39.98106
chr1:67686284..67686760	K562	HNRNPL	bodily fluid, blood	9.11210
chr1:67686526..67686870	prostate gland	POLR2A	prostate gland	30.63559
chr1:67686551..67687027	A549	NR3C1	lung	33.43418
chr1:67686566..67687010	A549	CEBPB	lung	37.83205
chr1:67686707..67686959	K562	ETV5	bodily fluid, blood	168.85727
chr1:67685754..67686973	HepG2	TAF1	endocrine gland, exocrine gland, liver, epithelium	41.95179
chr1:67686629..67687033	HEK293	ZNF623	kidney, epithelium	53.01161
chr1:67686329..67686819	transverse colon	CTCF	colon, large intestine, intestine	17.11666
chr1:67686613..67686917	HEK293	GLI2	kidney, epithelium	44.05803
chr1:67686637..67687053	MCF-7	CREB1	mammary gland, exocrine gland, epithelium	41.66799

chr1:67686453..67687077	A549	BCL3	lung	34.01783
chr1:67686590..67687023	gastroesophageal sphincter	POLR2A	musculature of body, stomach	284.64971
chr1:67686689..67686869	SK-N-SH	TAF1	brain	72.33601
chr1:67686750..67687046	K562	TEAD1	bodily fluid, blood	126.93155
chr1:67686244..67686868	HepG2	TIGD3	endocrine gland, exocrine gland, liver, epithelium	24.06211
chr1:67684758..67688906	K562	RBFOX2	bodily fluid, blood	462.77354
chr1:67684758..67688906	K562	RBFOX2	bodily fluid, blood	391.19249
chr1:67684758..67688906	K562	RBFOX2	bodily fluid, blood	320.14252
chr1:67684758..67688906	K562	RBFOX2	bodily fluid, blood	63.31824
chr1:67686671..67686854	HepG2	ATF3	endocrine gland, exocrine gland, liver, epithelium	92.96566
chr1:67686643..67687050	HEK293	ZNF843	kidney, epithelium	273.99319
chr1:67686337..67686781	HepG2	CENPT	endocrine gland, exocrine gland, liver, epithelium	27.88380
chr1:67686744..67687004	K562	JUNB	bodily fluid, blood	75.28524
chr1:67686650..67687086	SK-N-SH	TCF4	brain	65.96025
chr1:67686530..67686950	HepG2	ELF3	endocrine gland, exocrine gland, liver, epithelium	32.29260
chr1:67686674..67687244	GM12878	MTA2	bodily fluid, blood	42.37155
chr1:67686344..67686854	HepG2	ZNF746	endocrine gland, exocrine gland, liver, epithelium	42.30263
chr1:67686522..67687138	A549	JUN	lung	37.03437
chr1:67686728..67687344	A549	JUN	lung	13.17347
chr1:67686625..67686962	esophagus muscularis	POLR2A	esophagus, musculature of body	173.59777
chr1:67686617..67686998	body of pancreas	POLR2A	pancreas	292.28630
chr1:67686504..67686924	gastroesophageal sphincter	POLR2A	musculature of body, stomach	43.13932
chr1:67686515..67687031	HepG2	FOXJ3	endocrine gland, exocrine gland, liver, epithelium	27.89577
chr1:67686690..67687114	SK-N-SH	BNC2	brain	68.69446

chr1:67686468..67687204	HepG2	LIN54	endocrine gland, exocrine gland, liver, epithelium	35.36417
chr1:67686128..67686758	HepG2	ZBTB4	endocrine gland, exocrine gland, liver, epithelium	15.51168
chr1:67686731..67686935	A549	FOSL2	lung	222.83560
chr1:67686461..67686871	K562	REST	bodily fluid, blood	27.82145
chr1:67686686..67687041	HEK293	IKZF3	kidney, epithelium	278.51763
chr1:67686307..67687065	HEK293	ZNF189	kidney, epithelium	503.85253
chr1:67686116..67686796	K562	ZNF197	bodily fluid, blood	47.49400
chr1:67686528..67687208	K562	ZNF197	bodily fluid, blood	22.31364
chr1:67686685..67686848	HepG2	KLF16	endocrine gland, exocrine gland, liver, epithelium	59.62968
chr1:67686344..67686874	K562	MAZ	bodily fluid, blood	98.92402
chr1:67686177..67686827	HepG2	ZNF547	endocrine gland, exocrine gland, liver, epithelium	59.69148
chr1:67686749..67686950	A549	JUNB	lung	93.09954
chr1:67686682..67687066	K562	EGR1	bodily fluid, blood	66.00320
chr1:67686498..67686958	uterus	POLR2A	uterus	108.55390
chr1:67686528..67686938	HepG2	HOMEZ	endocrine gland, exocrine gland, liver, epithelium	47.20478
chr1:67686514..67686894	HepG2	RAD21	liver, epithelium, endocrine gland, exocrine gland	41.71480
chr1:67686579..67687034	Peyer's patch	POLR2A	intestine, lymphoid tissue	305.69775
chr1:67686648..67686894	GM12878	NBN	bodily fluid, blood	87.74452
chr1:67686391..67687011	body of pancreas	POLR2A	pancreas	530.23650
chr1:67686588..67687044	HEK293	ZNF398	kidney, epithelium	112.42537
chr1:67686574..67687078	A549	JUNB	lung	52.13345
chr1:67686688..67686898	breast epithelium	POLR2A	breast, epithelium	63.73715
chr1:67686475..67686991	K562	MAZ	bodily fluid, blood	53.17757
chr1:67686642..67686861	HepG2	RARA	endocrine gland, exocrine gland, liver, epithelium	58.03156

chr1:67686700..67686986	K562	TCF3	bodily fluid, blood	285.29266
chr1:67686534..67686910	lower leg skin	POLR2A	skin of body, limb	49.86030
chr1:67686603..67687033	GM12878	JUNB	bodily fluid, blood	39.90908
chr1:67686514..67687025	HEK293	PRDM10	kidney, epithelium	217.80422
chr1:67686465..67687005	K562	SMAD4	bodily fluid, blood	222.18958
chr1:67686665..67687205	K562	SMAD4	bodily fluid, blood	43.58864
chr1:67686584..67686980	GM12878	E2F8	bodily fluid, blood	36.96581
chr1:67686612..67687256	HEK293	TRIM28	kidney, epithelium	79.44843
chr1:67686454..67686958	HL-60	POLR2A	bodily fluid, blood	28.58537
chr1:67686652..67687096	A549	FOSL2	lung	32.41015
chr1:67686675..67687039	HeLa-S3	CHD2	uterus, epithelium	38.45488
chr1:67686688..67686958	A549	JUND	lung	21.13529
chr1:67685699..67687162	MCF 10A	POLR2A	mammary gland, exocrine gland, epithelium	148.10648
chr1:67686702..67686992	MCF-7	FOSL2	mammary gland, exocrine gland, epithelium	51.84929
chr1:67686637..67686897	Ishikawa	CEBPB	uterus	26.88462
chr1:67686392..67687028	K562	POLR2A	bodily fluid, blood	44.59674
chr1:67686672..67687342	HEK293	TRIM28	kidney, epithelium	32.80750
chr1:67686394..67686944	upper lobe of left lung	POLR2A	lung	102.75272
chr1:67686640..67687080	HEK293	ZNF121	kidney, epithelium	119.09479
chr1:67686621..67687005	thyroid gland	POLR2A	thyroid gland, endocrine gland	138.34246
chr1:67686621..67687085	K562	ELF4	bodily fluid, blood	81.32373
chr1:67686310..67686760	HepG2	ZNF205	endocrine gland, exocrine gland, liver, epithelium	40.47704
chr1:67686650..67686880	liver	ATF3	endocrine gland, exocrine gland, liver	137.42858
chr1:67686719..67686848	HepG2	HMG20A	endocrine gland, exocrine gland, liver, epithelium	44.40801

chr1:67686613..67687113	A549	JUNB	lung	73.68195
chr1:67686626..67686892	sigmoid colon	POLR2A	colon, large intestine, intestine	165.85102
chr1:67686153..67687044	tibial nerve	EP300	nerve, limb	141.30345
chr1:67686517..67686973	HepG2	MIER3	endocrine gland, exocrine gland, liver, epithelium	32.75976
chr1:67686532..67687062	A549	JUNB	lung	41.49276
chr1:67686661..67686999	sigmoid colon	POLR2A	colon, large intestine, intestine	100.53216
chr1:67686653..67687057	HEK293	ZNF324	kidney, epithelium	65.15673
chr1:67686459..67686959	HepG2	ATF7	endocrine gland, exocrine gland, liver, epithelium	32.79305
chr1:67686711..67687091	K562	NCOA2	bodily fluid, blood	54.97581
chr1:67686471..67687015	HeLa-S3	POLR2A	uterus, epithelium	25.79818
chr1:67686671..67687215	HeLa-S3	POLR2A	uterus, epithelium	4.40477
chr1:67686723..67687007	MCF 10A	FOS	mammary gland, exocrine gland, epithelium	173.60316
chr1:67686617..67686893	MCF-7	CEBPB	mammary gland, exocrine gland, epithelium	31.07292
chr1:67686480..67686896	K562	MTA2	bodily fluid, blood	59.75045
chr1:67686705..67687121	K562	MTA2	bodily fluid, blood	52.39359
chr1:67686337..67687207	K562	ZNF274	bodily fluid, blood	32.99309
chr1:67686091..67686961	K562	ZNF274	bodily fluid, blood	30.73524
chr1:67685642..67687123	IMR-90	POLR2A	lung, connective tissue	99.12295
chr1:67686694..67687004	H1	JUND	embryo	18.28033
chr1:67686676..67687136	HeLa-S3	STAT1	uterus, epithelium	18.49585
chr1:67686692..67686942	HepG2	JUND	endocrine gland, exocrine gland, liver, epithelium	53.73819
chr1:67686573..67687037	K562	E2F8	bodily fluid, blood	39.70624
chr1:67686566..67687080	SK-N-SH	POLR2A	brain	119.50492
chr1:67686685..67686947	A549	NR3C1	lung	149.82680

chr1:67686633..67686926	A549	JUN	lung	35.72370
chr1:67686731..67686995	K562	USF1	bodily fluid, blood	16.26766
chr1:67686557..67686997	A549	SMC3	lung	32.63397
chr1:67686728..67687228	A549	EP300	lung	13.36771
chr1:67684910..67687139	A549	JUN	lung	210.24207
chr1:67684910..67687139	A549	JUN	lung	154.15954
chr1:67684910..67687139	A549	JUN	lung	145.48092
chr1:67686338..67686762	K562	CTCF	bodily fluid, blood	12.22328
chr1:67686565..67686989	K562	CTCF	bodily fluid, blood	10.23528
chr1:67686599..67686929	HepG2	ZNF12	endocrine gland, exocrine gland, liver, epithelium	21.79993
chr1:67686299..67686989	HepG2	POLR2A	endocrine gland, exocrine gland, liver, epithelium	89.57046
chr1:67686427..67686907	HepG2	ZNF124	endocrine gland, exocrine gland, liver, epithelium	35.54806
chr1:67684983..67687391	A549	JUN	lung	46.65191
chr1:67684983..67687391	A549	JUN	lung	103.21651
chr1:67685726..67687073	A549	JUN	lung	58.68361
chr1:67686552..67686992	HeLa-S3	ELK4	uterus, epithelium	20.44994
chr1:67686708..67686837	HepG2	HDAC2	endocrine gland, exocrine gland, liver, epithelium	57.44879
chr1:67686533..67686997	HepG2	ZBTB43	endocrine gland, exocrine gland, liver, epithelium	49.44581
chr1:67686585..67686983	HepG2	FOSL2	endocrine gland, exocrine gland, liver, epithelium	238.79023
chr1:67686341..67686941	K562	HMG20A	bodily fluid, blood	44.83003
chr1:67686316..67686946	A549	SIN3A	lung	40.43297
chr1:67686635..67687065	A549	FOSL2	lung	57.49187
chr1:67686258..67686798	HepG2	ZNF274	endocrine gland, exocrine gland, liver, epithelium	36.85710
chr1:67686537..67686885	BLaER1	CEBPA	bone marrow, bone element	5.20174

chr1:67686542..67686852	GM12878	RELA	bodily fluid, blood	42.69462
chr1:67684895..67687089	A549	JUN	lung	98.36590
chr1:67686402..67686786	neural cell	POLR2A		30.74444
chr1:67686645..67687089	A549	SMC3	lung	38.99628
chr1:67686620..67686855	HeLa-S3	POLR2A	uterus, epithelium	46.55129
chr1:67686651..67686935	H1	MAFK	embryo	29.01943
chr1:67686463..67686928	K562	POLR2H	bodily fluid, blood	169.86097
chr1:67686439..67687095	HepG2	ZNF221	endocrine gland, exocrine gland, liver, epithelium	53.15017
chr1:67686540..67686980	HepG2	ATF4	endocrine gland, exocrine gland, liver, epithelium	138.26583
chr1:67686650..67686986	A549	EP300	lung	165.74947
chr1:67686601..67687001	HepG2	MXI1	endocrine gland, exocrine gland, liver, epithelium	48.29550
chr1:67686708..67686959	K562	NFE2	bodily fluid, blood	231.56576
chr1:67686399..67686879	GM12878	POLR2A	bodily fluid, blood	34.06759
chr1:67686620..67686980	K562	ZNF24	bodily fluid, blood	78.09680
chr1:67686596..67686860	liver	RXRA	endocrine gland, exocrine gland, liver	131.02747
chr1:67686726..67686982	IMR-90	USF2	lung, connective tissue	19.76576
chr1:67686377..67686817	SK-N-SH	TFAP2B	brain	122.37133
chr1:67686581..67687021	SK-N-SH	TFAP2B	brain	59.60845
chr1:67686652..67687012	K562	SMAD1	bodily fluid, blood	20.29562
chr1:67686527..67686927	HepG2	PPARG	endocrine gland, exocrine gland, liver, epithelium	58.32349
chr1:67686485..67686941	breast epithelium	POLR2A	breast, epithelium	41.14086
chr1:67686653..67687062	K562	DPF2	bodily fluid, blood	250.44550
chr1:67686614..67686884	HCT116	YY1	colon, large intestine, epithelium, intestine	18.63524
chr1:67686655..67687035	A549	FOSL2	lung	27.51775

chr1:67686725..67686945	A549	NR3C1	lung	86.78531
chr1:67686540..67687010	A549	RAD21	lung	15.97823
chr1:67686485..67687055	A549	BCL3	lung	43.20543
chr1:67686249..67686819	A549	BCL3	lung	36.78798
chr1:67686644..67686944	A549	NR3C1	lung	22.69602
chr1:67686652..67686996	K562	RCOR1	bodily fluid, blood	41.21782
chr1:67686597..67687021	K562	USF1	bodily fluid, blood	69.36067
chr1:67686753..67686913	HeLa-S3	EP300	uterus, epithelium	78.23811
chr1:67686625..67686978	stomach	POLR2A	stomach	231.98262
chr1:67686399..67686795	esophagus squamous	CTCF	epithelium, esophagus	30.84143
chr1:67686532..67687310	K562	DPF2	bodily fluid, blood	558.19305
chr1:67686648..67687051	vagina	POLR2A	vagina	144.04617
chr1:67686634..67687050	Ishikawa	EP300	uterus	33.04183
chr1:67686370..67686870	heart right ventricle	CTCF	heart	27.95781
chr1:67686595..67687085	SK-N-MC	POLR2A	epithelium, brain	38.70890
chr1:67686572..67687096	A549	MAX	lung	26.57451
chr1:67686367..67686891	A549	MAX	lung	44.72840
chr1:67686648..67687104	A549	SMC3	lung	39.42965
chr1:67686481..67686917	GM15510	POLR2A	bodily fluid, blood	35.89885
chr1:67686675..67686942	A549	NR3C1	lung	144.67516
chr1:67686628..67686952	HepG2	MAX	endocrine gland, exocrine gland, liver, epithelium	27.70456
chr1:67686315..67686899	HepG2	MXD4	endocrine gland, exocrine gland, liver, epithelium	34.82555
chr1:67686693..67686949	HepG2	NFE2L2	endocrine gland, exocrine gland, liver, epithelium	44.52528
chr1:67686598..67687048	HepG2	GABPA	endocrine gland, exocrine gland, liver, epithelium	52.54251

chr1:67686515..67686999	K562	ELF1	bodily fluid, blood	63.61168
chr1:67686569..67687053	GM12878	EED	bodily fluid, blood	51.75722
chr1:67686436..67687128	transverse colon	POLR2A	colon, large intestine, intestine	287.82026
chr1:67686155..67686859	HepG2	UBTF	endocrine gland, exocrine gland, liver, epithelium	64.82358
chr1:67686717..67687030	A549	JUN	lung	89.19750
chr1:67686533..67686869	HepG2	HDAC2	endocrine gland, exocrine gland, liver, epithelium	28.86781
chr1:67686676..67686946	HepG2	SMC3	endocrine gland, exocrine gland, liver, epithelium	35.62408
chr1:67686464..67686784	H1	CREB1	embryo	28.77997
chr1:67686052..67686892	C4-2B	CTCF	prostate gland	79.98583
chr1:67686176..67687021	HeLa-S3	POLR2A	uterus, epithelium	74.26071
chr1:67686176..67687021	HeLa-S3	POLR2A	uterus, epithelium	40.05777
chr1:67686423..67686933	middle frontal area 46	CTCF	brain	19.27005
chr1:67686709..67686908	K562	MAX	bodily fluid, blood	86.43996
chr1:67686572..67686916	liver	HNF4A	endocrine gland, exocrine gland, liver	277.36828
chr1:67686270..67686866	HepG2	SMAD1	endocrine gland, exocrine gland, liver, epithelium	12.17684
chr1:67686176..67686756	HepG2	KMT2A	endocrine gland, exocrine gland, liver, epithelium	56.29264
chr1:67686611..67687015	HEK293	KLF16	kidney, epithelium	55.69128
chr1:67686562..67687038	MCF-7	SIN3A	mammary gland, exocrine gland, epithelium	53.13663
chr1:67686529..67686949	HepG2	ZBTB26	endocrine gland, exocrine gland, liver, epithelium	44.13959
chr1:67686538..67686978	HEK293	ZSCAN21	kidney, epithelium	110.49297
chr1:67686741..67687181	HEK293	ZSCAN21	kidney, epithelium	19.70071
chr1:67686605..67687069	K562	NR4A1	bodily fluid, blood	55.91092
chr1:67686672..67686976	MCF 10A	MYC	mammary gland, exocrine gland, epithelium	37.85277
chr1:67686581..67686871	HepG2	HNF4A	endocrine gland, exocrine gland, liver, epithelium	24.81387

chr1:67686473..67686923	HepG2	ZNF263	endocrine gland, exocrine gland, liver, epithelium	15.34367
chr1:67686713..67686825	HepG2	ATF2	endocrine gland, exocrine gland, liver, epithelium	38.94752
chr1:67686670..67687086	K562	GTF2F1	bodily fluid, blood	75.61623
chr1:67686655..67687051	K562	TCF12	bodily fluid, blood	116.74186
chr1:67686552..67686912	HepG2	HNF4A	endocrine gland, exocrine gland, liver, epithelium	100.30427
chr1:67686512..67686936	HepG2	MYNN	endocrine gland, exocrine gland, liver, epithelium	30.67151
chr1:67686441..67686861	HepG2	ELF1	endocrine gland, exocrine gland, liver, epithelium	44.62002
chr1:67686650..67687070	HepG2	ELF1	endocrine gland, exocrine gland, liver, epithelium	18.02696
chr1:67686662..67686895	HepG2	TP53	endocrine gland, exocrine gland, liver, epithelium	304.43531
chr1:67686645..67687061	K562	MBD2	bodily fluid, blood	39.47080
chr1:67686664..67687064	MCF-7	JUND	mammary gland, exocrine gland, epithelium	24.87821
chr1:67686652..67686942	HepG2	REST	endocrine gland, exocrine gland, liver, epithelium	27.12454
chr1:67685583..67686875	GM12878	POLR2A	bodily fluid, blood	75.40443
chr1:67685583..67686875	GM12878	POLR2A	bodily fluid, blood	37.17262
chr1:67685583..67686875	GM12878	POLR2A	bodily fluid, blood	34.77816
chr1:67686330..67686800	A549	ZBTB33	lung	34.69591
chr1:67686552..67687002	HepG2	ZNF264	endocrine gland, exocrine gland, liver, epithelium	36.50616
chr1:67686491..67687018	HepG2	SAP130	endocrine gland, exocrine gland, liver, epithelium	106.91398
chr1:67686700..67687100	MCF-7	DPF2	mammary gland, exocrine gland, epithelium	57.11533
chr1:67686503..67686811	HEK293	ZNF2	kidney, epithelium	140.60007
chr1:67686734..67686972	HEK293	ZNF2	kidney, epithelium	86.30990
chr1:67686652..67686928	HepG2	MAFF	endocrine gland, exocrine gland, liver, epithelium	54.95607
chr1:67686374..67687144	erythroblast	POLR2A	bone marrow, bone element	374.63291
chr1:67686737..67686937	HepG2	USF1	endocrine gland, exocrine gland, liver, epithelium	16.48371

chr1:67686599..67686919	A549	CEBPB	lung	27.81492
chr1:67686529..67687025	HCT116	MAX	colon, large intestine, epithelium, intestine	132.71517
chr1:67686735..67686880	GM12878	MLLT1	bodily fluid, blood	64.61975
chr1:67686474..67686844	HepG2	ZBTB21	endocrine gland, exocrine gland, liver, epithelium	25.15812
chr1:67686591..67686997	K562	ATF7	bodily fluid, blood	200.66213
chr1:67686590..67686914	uterus	POLR2A	uterus	41.54235
chr1:67686593..67686963	HCT116	ATF3	colon, large intestine, epithelium, intestine	97.84619
chr1:67686485..67687009	liver	GABPA	endocrine gland, exocrine gland, liver	12.22561
chr1:67686580..67686900	sigmoid colon	POLR2A	colon, large intestine, intestine	45.47278
chr1:67686714..67687014	K562	PML	bodily fluid, blood	95.04767
chr1:67686543..67687031	GM12878	IKZF1	bodily fluid, blood	496.89282
chr1:67686690..67687068	SK-N-SH	GATA3	brain	399.89570
chr1:67686624..67686908	HepG2	FOXA1	endocrine gland, exocrine gland, liver, epithelium	31.77371
chr1:67686554..67687050	GM18951	POLR2A	bodily fluid, blood	13.59374
chr1:67686720..67686935	HCT116	SP1	colon, large intestine, epithelium, intestine	185.95535
chr1:67686541..67686997	liver	MAX	endocrine gland, exocrine gland, liver	51.27688
chr1:67686084..67686854	HepG2	ZFP91	endocrine gland, exocrine gland, liver, epithelium	83.65914
chr1:67686546..67686962	K562	POLR2A	bodily fluid, blood	57.03109
chr1:67686748..67687164	K562	POLR2A	bodily fluid, blood	21.55258
chr1:67686401..67686831	GM18526	POLR2A	bodily fluid, blood	32.83243
chr1:67686670..67686860	HepG2	CEBPA	endocrine gland, exocrine gland, liver, epithelium	111.63165
chr1:67686749..67686989	stomach	POLR2A	stomach	75.68787
chr1:67686507..67686883	K562	ZNF184	bodily fluid, blood	39.48128
chr1:67686596..67687072	HepG2	ZKSCAN8	endocrine gland, exocrine gland, liver, epithelium	33.40964

chr1:67686476..67686860	MCF-7	POLR2A	mammary gland, exocrine gland, epithelium	65.55568
chr1:67686703..67686967	K562	FOS	bodily fluid, blood	38.26926
chr1:67686585..67686914	liver	HNF4A	endocrine gland, exocrine gland, liver	225.49377
chr1:67686711..67686991	K562	JUND	bodily fluid, blood	265.24250
chr1:67686477..67686827	HepG2	KDM2A	endocrine gland, exocrine gland, liver, epithelium	58.58148
chr1:67686677..67686993	K562	NONO	bodily fluid, blood	45.29146
chr1:67686744..67686988	HeLa-S3	FOS	uterus, epithelium	31.29933
chr1:67686723..67687019	K562	ZNF384	bodily fluid, blood	22.74443
chr1:67686636..67687092	HCT116	SRF	colon, large intestine, epithelium, intestine	42.59638
chr1:67686565..67687035	HepG2	ZFP1	endocrine gland, exocrine gland, liver, epithelium	15.71004
chr1:67686638..67686974	IMR-90	MXI1	lung, connective tissue	50.10079
chr1:67686744..67686979	K562	NR2F2	bodily fluid, blood	104.00606
chr1:67686234..67686930	HepG2	ZGPAT	endocrine gland, exocrine gland, liver, epithelium	60.28640
chr1:67684983..67687391	A549	JUN	lung	83.78075
chr1:67686469..67686779	K562	ELF4	bodily fluid, blood	54.35899
chr1:67686625..67687034	prostate gland	POLR2A	prostate gland	237.45489
chr1:67686341..67686801	A549	RAD21	lung	67.96411
chr1:67686554..67687154	K562	GMEB1	bodily fluid, blood	152.96627
chr1:67686654..67686886	HepG2	GATAD2A	endocrine gland, exocrine gland, liver, epithelium	75.28908
chr1:67686550..67686994	A549	RAD21	lung	36.44667
chr1:67686516..67687100	HepG2	MXD4	endocrine gland, exocrine gland, liver, epithelium	18.10058
chr1:67686690..67686834	SK-N-SH	MXI1	brain	74.66408
chr1:67686575..67686845	BLaER1	CEBPA	bone marrow, bone element	2.56549
chr1:67684960..67686930	K562	SUPT5H	bodily fluid, blood	197.37555

chr1:67684960..67686930	K562	SUPT5H	bodily fluid, blood	66.69510
chr1:67684960..67686930	K562	SUPT5H	bodily fluid, blood	1047.14735
chr1:67686298..67686898	A549	JUN	lung	57.86874
chr1:67686239..67686883	HepG2	ZNF790	endocrine gland, exocrine gland, liver, epithelium	31.41227
chr1:67686676..67686958	K562	HDAC1	bodily fluid, blood	143.03892
chr1:67686532..67687310	K562	DPF2	bodily fluid, blood	90.26703
chr1:67686749..67686892	A549	EP300	lung	79.08106
chr1:67686622..67687122	A549	JUNB	lung	48.22962
chr1:67686616..67686940	HepG2	SP1	endocrine gland, exocrine gland, liver, epithelium	17.05259
chr1:67686617..67687053	A549	SMC3	lung	55.86930
chr1:67686680..67686932	A549	NR3C1	lung	129.48499
chr1:67686553..67686793	A549	JUN	lung	75.55411
chr1:67684910..67687139	A549	JUN	lung	496.45467
chr1:67686385..67686789	A549	RAD21	lung	22.46803
chr1:67686468..67686912	MCF-7	MNT	mammary gland, exocrine gland, epithelium	62.39689
chr1:67686645..67686958	A549	NR3C1	lung	150.81050
chr1:67686593..67686857	GM12878	EBF1	bodily fluid, blood	35.85257
chr1:67686690..67686969	K562	JUND	bodily fluid, blood	185.78158
chr1:67686695..67686922	K562	MYC	bodily fluid, blood	103.95787
chr1:67686343..67686807	A549	RAD21	lung	74.05057
chr1:67686544..67687008	A549	RAD21	lung	46.69733
chr1:67686674..67686933	A549	EP300	lung	97.88431
chr1:67686418..67687022	K562	ZNF766	bodily fluid, blood	54.53472
chr1:67684943..67687064	HepG2	POLR2G	endocrine gland, exocrine gland, liver, epithelium	53.43169

chr1:67684943..67687064	HepG2	POLR2G	endocrine gland, exocrine gland, liver, epithelium	52.61030
chr1:67686573..67687217	K562	ELF1	bodily fluid, blood	43.26532
chr1:67686271..67686767	HepG2	KDM3A	endocrine gland, exocrine gland, liver, epithelium	36.02837
chr1:67686627..67687047	A549	GATA3	lung	54.31709
chr1:67686518..67686999	K562	ATF4	bodily fluid, blood	689.56250
chr1:67686522..67686992	H1	POLR2A	embryo	16.40827
chr1:67686587..67686951	HepG2	EP300	endocrine gland, exocrine gland, liver, epithelium	62.26447
chr1:67686482..67686782	neural cell	MXI1		103.15799
chr1:67686534..67687138	neural cell	MXI1		19.27590
chr1:67686744..67687068	K562	MXI1	bodily fluid, blood	25.69335
chr1:67686603..67687033	A549	FOSL2	lung	28.02939
chr1:67686668..67687052	K562	ZBTB5	bodily fluid, blood	44.69944
chr1:67686703..67687023	HEK293	ZNF660	kidney, epithelium	151.20057
chr1:67686194..67687034	GM12878	POLR2A	bodily fluid, blood	82.41773
chr1:67686194..67687034	GM12878	POLR2A	bodily fluid, blood	48.15994
chr1:67686640..67686970	HeLa-S3	TAF1	uterus, epithelium	50.54251
chr1:67686659..67687039	Ishikawa	NFIC	uterus	52.45441
chr1:67686602..67686966	HepG2	TBL1XR1	endocrine gland, exocrine gland, liver, epithelium	26.20744
chr1:67686517..67687001	K562	ARNT	bodily fluid, blood	60.79126
chr1:67686479..67687113	K562	ATF3	bodily fluid, blood	1069.68789
chr1:67686405..67686805	HepG2	PATZ1	endocrine gland, exocrine gland, liver, epithelium	32.40500
chr1:67686515..67686999	K562	POLR2A	bodily fluid, blood	29.77570
chr1:67686468..67686824	K562	TOE1	bodily fluid, blood	38.01964
chr1:67686579..67687059	A549	FOSL2	lung	55.32916

chr1:67686667..67686997	HeLa-S3	MAX	uterus, epithelium	27.25597
chr1:67686545..67687044	K562	HDAC1	bodily fluid, blood	233.63071
chr1:67686653..67687157	HEK293	ZNF501	kidney, epithelium	45.76579
chr1:67686535..67686821	BLaER1	CEBPA	bone marrow, bone element	4.23583
chr1:67686643..67686867	HepG2	ATF3	endocrine gland, exocrine gland, liver, epithelium	21.33532
chr1:67686521..67686965	K562	CC2D1A	bodily fluid, blood	56.98484
chr1:67686722..67687166	K562	CC2D1A	bodily fluid, blood	31.48831
chr1:67686554..67686910	HepG2	ETS1	endocrine gland, exocrine gland, liver, epithelium	34.04089
chr1:67686560..67687211	K562	FOSL1	bodily fluid, blood	618.11252
chr1:67686601..67686971	HepG2	EP300	endocrine gland, exocrine gland, liver, epithelium	22.73850
chr1:67686519..67686943	K562	CCNT2	bodily fluid, blood	47.19154
chr1:67686651..67687221	GM12878	IKZF1	bodily fluid, blood	45.47134
chr1:67686569..67686964	liver	RXRA	endocrine gland, exocrine gland, liver	243.18018
chr1:67686692..67686968	HeLa-S3	MAFF	uterus, epithelium	55.45166
chr1:67686628..67686872	HepG2	HLF	endocrine gland, exocrine gland, liver, epithelium	45.05785
chr1:67686631..67686887	GM12878	TCF12	bodily fluid, blood	20.71260
chr1:67686258..67686998	K562	ZNF589	bodily fluid, blood	46.12330
chr1:67686558..67686982	A549	CEBPB	lung	34.51338
chr1:67686404..67686784	HepG2	ZC3H4	endocrine gland, exocrine gland, liver, epithelium	21.02682
chr1:67686572..67687076	GM12878	SIN3A	bodily fluid, blood	36.13461
chr1:67686653..67687042	K562	ZNF318	bodily fluid, blood	275.34726
chr1:67686653..67687042	K562	ZNF318	bodily fluid, blood	10.22120
chr1:67686712..67686989	MCF 10A	FOS	mammary gland, exocrine gland, epithelium	159.96347
chr1:67686489..67687053	HCT116	SIN3A	colon, large intestine, epithelium, intestine	82.27660

chr1:67686621..67687051	HepG2	TEAD4	endocrine gland, exocrine gland, liver, epithelium	33.36476
chr1:67686728..67687001	MCF 10A	FOS	mammary gland, exocrine gland, epithelium	141.16962
chr1:67686596..67686986	K562	MYC	bodily fluid, blood	24.33677
chr1:67686226..67686902	K562	KAT7	bodily fluid, blood	61.47356
chr1:67686605..67687009	K562	POLR2A	bodily fluid, blood	77.29105
chr1:67686680..67686936	HepG2	FOSL2	endocrine gland, exocrine gland, liver, epithelium	100.79313
chr1:67686638..67686894	K562	SIN3A	bodily fluid, blood	42.23496
chr1:67686718..67687008	K562	SIRT6	bodily fluid, blood	24.90331
chr1:67686447..67686983	endothelial cell of umbilical vein	POLR2A	blood vessel, vein, placenta, extraembryonic component, vasculature, epithelium	46.98756
chr1:67686620..67686778	HepG2	POLR2A	endocrine gland, exocrine gland, liver, epithelium	68.73753
chr1:67686262..67686806	K562	LCOR	bodily fluid, blood	52.47189
chr1:67686465..67687009	K562	LCOR	bodily fluid, blood	39.98760
chr1:67686443..67686847	K562	NR2C2	bodily fluid, blood	57.94870
chr1:67686709..67686999	HeLa-S3	USF2	uterus, epithelium	49.97410
chr1:67686498..67687062	K562	SIN3A	bodily fluid, blood	30.13465
chr1:67686661..67686977	K562	HCFC1	bodily fluid, blood	17.35449
chr1:67686498..67687148	HeLa-S3	SMARCC1	uterus, epithelium	47.73055
chr1:67686466..67686930	K562	ESRRA	bodily fluid, blood	61.91820
chr1:67686615..67687147	erythroblast	GATA1	bone marrow, bone element	179.67989
chr1:67686615..67687169	erythroblast	GATA1	bone marrow, bone element	83.69903
chr1:67686732..67686902	HepG2	KDM1A	endocrine gland, exocrine gland, liver, epithelium	76.88641
chr1:67686689..67686913	Ishikawa	RAD21	uterus	19.40521
chr1:67686477..67686837	K562	ZFP91	bodily fluid, blood	51.12705
chr1:67686589..67686959	K562	NCOR1	bodily fluid, blood	31.29324

chr1:67686689..67686929	IMR-90	NFE2L2	lung, connective tissue	51.34049
chr1:67686364..67686920	HepG2	ZSCAN25	endocrine gland, exocrine gland, liver, epithelium	80.57651
chr1:67686741..67686936	IMR-90	FOS	lung, connective tissue	102.93552
chr1:67686366..67686850	HepG2	MAX	endocrine gland, exocrine gland, liver, epithelium	54.10929
chr1:67686643..67686993	GM12878	BCL3	bodily fluid, blood	19.37019
chr1:67685038..67687053	K562	POLR2A	bodily fluid, blood	410.53970
chr1:67685038..67687053	K562	POLR2A	bodily fluid, blood	100.04355
chr1:67686370..67686874	K562	HDAC1	bodily fluid, blood	35.55289
chr1:67686317..67686761	K562	SETDB1	bodily fluid, blood	6.32509
chr1:67686637..67687037	HEK293	OSR2	kidney, epithelium	410.62047
chr1:67686633..67686977	K562	MYC	bodily fluid, blood	27.03613
chr1:67686693..67687057	SK-N-SH	RCOR1	brain	65.76875
chr1:67686384..67686954	HepG2	POGK	endocrine gland, exocrine gland, liver, epithelium	48.74348
chr1:67686548..67686968	liver	NR2F2	endocrine gland, exocrine gland, liver	26.55222
chr1:67686314..67686844	neural cell	SMC3		96.58909
chr1:67686366..67686914	A549	SIN3A	lung	87.03755
chr1:67686672..67687056	K562	JUNB	bodily fluid, blood	68.44476
chr1:67686390..67687000	K562	ZNF24	bodily fluid, blood	57.62345
chr1:67686665..67686995	GM12878	BHLHE40	bodily fluid, blood	41.45728
chr1:67686735..67686991	SK-N-SH	REST	brain	39.03969
chr1:67686701..67686941	SK-N-SH	SMC3	brain	20.42102
chr1:67686628..67687021	K562	POLR2A	bodily fluid, blood	50.03890
chr1:67686549..67686905	prostate gland	POLR2A	prostate gland	36.88988
chr1:67686389..67686765	HepG2	YY1	endocrine gland, exocrine gland, liver, epithelium	27.43570

chr1:67686377..67686853	HepG2	POLR2A	endocrine gland, exocrine gland, liver, epithelium	47.00803
chr1:67686671..67686921	BLaER1	CEBPA	bone marrow, bone element	3.10416
chr1:67686261..67686911	H1	CHD1	embryo	39.10832
chr1:67686744..67687334	GM12878	NFIC	bodily fluid, blood	9.65388
chr1:67686603..67687079	liver	JUND	endocrine gland, exocrine gland, liver	49.69497
chr1:67686629..67686933	GM12878	PAX5	bodily fluid, blood	29.58523
chr1:67686297..67686873	HepG2	IRF9	endocrine gland, exocrine gland, liver, epithelium	37.16321
chr1:67686222..67686818	GM12878	DPF2	bodily fluid, blood	71.76977
chr1:67686618..67687002	HeLa-S3	MAX	uterus, epithelium	80.61210
chr1:67686548..67686912	suprapubic skin	POLR2A	skin of body	38.06893
chr1:67685642..67687123	IMR-90	POLR2A	lung, connective tissue	62.18141
chr1:67686426..67686942	HCT116	POLR2A	colon, large intestine, epithelium, intestine	10.85090
chr1:67686746..67686963	K562	NFE2	bodily fluid, blood	238.93542
chr1:67686277..67687028	HCT116	POLR2A	colon, large intestine, epithelium, intestine	138.40273
chr1:67686655..67686935	A549	NR3C1	lung	31.00835
chr1:67686484..67686974	HEK293	ZBTB11	kidney, epithelium	40.93244
chr1:67686706..67687006	HEK293	INSM2	kidney, epithelium	205.87029
chr1:67686665..67686840	IMR-90	CEBPB	lung, connective tissue	110.92914
chr1:67686559..67686849	HepG2	HNF4G	endocrine gland, exocrine gland, liver, epithelium	45.15104
chr1:67686562..67686818	A549	CEBPB	lung	22.81100
chr1:67685072..67686828	HepG2	AGO2	endocrine gland, exocrine gland, liver, epithelium	185.26505
chr1:67685072..67686828	HepG2	AGO2	endocrine gland, exocrine gland, liver, epithelium	137.15704
chr1:67686676..67687000	K562	SMARCA4	bodily fluid, blood	173.02207
chr1:67686654..67686894	HepG2	MAFK	endocrine gland, exocrine gland, liver, epithelium	36.49087

chr1:67686729..67686919	endothelial cell of umbilical vein	MYC	blood vessel, vein, placenta, extraembryonic component, vasculature, epithelium	27.03325
chr1:67686668..67687004	HeLa-S3	JUN	uterus, epithelium	39.48517
chr1:67686608..67686888	H54	POLR2A	brain	25.39434
chr1:67686655..67687039	MCF-7	HDAC2	mammary gland, exocrine gland, epithelium	14.95658
chr1:67686660..67687024	GM12878	BACH1	bodily fluid, blood	52.48592
chr1:67686737..67687017	K562	JUN	bodily fluid, blood	42.99959
chr1:67686750..67686954	K562	NFE2	bodily fluid, blood	11.85759
chr1:67686470..67687140	HEK293	TRIM28	kidney, epithelium	100.72189
chr1:67686433..67687176	HEK293	SP7	kidney, epithelium	561.76221
chr1:67686652..67686982	K562	YY1	bodily fluid, blood	19.93759
chr1:67686605..67687197	K562	IKZF1	bodily fluid, blood	18.78145
chr1:67686605..67687197	K562	IKZF1	bodily fluid, blood	619.21385
chr1:67686394..67686764	heart left ventricle	CTCF	heart	28.02998
chr1:67686685..67687055	heart left ventricle	CTCF	heart	7.20996
chr1:67686678..67687078	SK-N-SH	GABPA	brain	38.07895
chr1:67686595..67687145	upper lobe of left lung	POLR2A	lung	10.66602
chr1:67686634..67687104	Ishikawa	FOXM1	uterus	19.09421
chr1:67686611..67687055	K562	MTA1	bodily fluid, blood	86.06624
chr1:67686718..67686968	A549	RAD21	lung	13.43922
chr1:67686684..67687100	K562	PBX2	bodily fluid, blood	60.69578
chr1:67686690..67687030	K562	MLLT1	bodily fluid, blood	25.07492
chr1:67686679..67686989	HeLa-S3	MAFK	uterus, epithelium	53.23882
chr1:67686562..67686906	breast epithelium	POLR2A	breast, epithelium	34.63585
chr1:67686694..67686930	K562	ATF3	bodily fluid, blood	96.56635

chr1:67686482..67686852	HepG2	TEAD3	endocrine gland, exocrine gland, liver, epithelium	78.90189
chr1:67686595..67686819	HepG2	CEBPB	endocrine gland, exocrine gland, liver, epithelium	27.27137
chr1:67686550..67687040	GM12878	CBFB	bodily fluid, blood	39.54262
chr1:67686326..67686862	HepG2	TCF12	endocrine gland, exocrine gland, liver, epithelium	24.64544
chr1:67686673..67687003	HEK293T	CTBP1	kidney, epithelium	74.79689
chr1:67686706..67686946	transverse colon	EP300	colon, large intestine, intestine	26.09339
chr1:67686465..67687145	K562	ZNF83	bodily fluid, blood	25.68204
chr1:67686691..67686851	HepG2	CEBPG	endocrine gland, exocrine gland, liver, epithelium	89.86566
chr1:67686228..67686828	HepG2	HNRNPLL	endocrine gland, exocrine gland, liver, epithelium	23.89169
chr1:67686304..67687048	HepG2	ARID4A	endocrine gland, exocrine gland, liver, epithelium	129.64315
chr1:67686245..67686941	HepG2	ZNF784	endocrine gland, exocrine gland, liver, epithelium	59.86938
chr1:67686691..67687341	A549	JUN	lung	52.27045
chr1:67686594..67687070	A549	JUNB	lung	57.51396
chr1:67686570..67686999	K562	NR2F1	bodily fluid, blood	143.29317
chr1:67686570..67686999	K562	NR2F1	bodily fluid, blood	68.62448
chr1:67686468..67686854	K562	POLR2B	bodily fluid, blood	128.69619
chr1:67686564..67687068	A549	JUNB	lung	26.03391
chr1:67686581..67687179	K562	SMARCA4	bodily fluid, blood	423.66867
chr1:67686315..67686771	GM12878	ZNF687	bodily fluid, blood	55.35440
chr1:67684910..67690102	HepG2	RBFOX2	endocrine gland, exocrine gland, liver, epithelium	385.22897
chr1:67684910..67690102	HepG2	RBFOX2	endocrine gland, exocrine gland, liver, epithelium	261.84912
chr1:67684910..67690102	HepG2	RBFOX2	endocrine gland, exocrine gland, liver, epithelium	231.93019
chr1:67684910..67690102	HepG2	RBFOX2	endocrine gland, exocrine gland, liver, epithelium	141.02217
chr1:67684910..67690102	HepG2	RBFOX2	endocrine gland, exocrine gland, liver, epithelium	108.08166

chr1:67684910..67690102	HepG2	RBFOX2	endocrine gland, exocrine gland, liver, epithelium	74.95649
chr1:67686274..67686836	HEK293	ZBTB20	kidney, epithelium	115.42128
chr1:67686589..67686994	A549	EP300	lung	212.77009
chr1:67686599..67687155	PFSK-1	POLR2A	connective tissue, brain	46.94028
chr1:67686560..67686924	K562	RAD21	bodily fluid, blood	77.03382
chr1:67686669..67686972	K562	SMARCC2	bodily fluid, blood	173.78430
chr1:67686654..67687074	HepG2	TFE3	endocrine gland, exocrine gland, liver, epithelium	25.17475
chr1:67686538..67687022	HepG2	KLF11	endocrine gland, exocrine gland, liver, epithelium	32.26581
chr1:67686744..67687228	HepG2	KLF11	endocrine gland, exocrine gland, liver, epithelium	21.09086
chr1:67686710..67687026	K562	CBFA2T2	bodily fluid, blood	569.10063
chr1:67686575..67686985	K562	NR2C1	bodily fluid, blood	41.34683
chr1:67686721..67687035	K562	HDAC2	bodily fluid, blood	198.25001
chr1:67686493..67686869	ovary	POLR2A	ovary, gonad	28.27268
chr1:67686524..67686874	HepG2	CTCF	endocrine gland, exocrine gland, liver, epithelium	16.82717
chr1:67686326..67686802	gastrocnemius medialis	CTCF	musculature of body, limb	36.05339
chr1:67686445..67686925	HepG2	ZNF217	endocrine gland, exocrine gland, liver, epithelium	39.89086
chr1:67686696..67686912	HepG2	RAD21	endocrine gland, exocrine gland, liver, epithelium	37.64999
chr1:67686439..67687059	adrenal gland	POLR2A	endocrine gland, adrenal gland	688.97309
chr1:67686433..67686953	HEK293	SCRT2	kidney, epithelium	83.80512
chr1:67686491..67686847	gastrocnemius medialis	POLR2A	musculature of body, limb	209.30717
chr1:67686463..67687033	GM12878	MTA2	bodily fluid, blood	131.31771
chr1:67686613..67687029	K562	PRDM10	bodily fluid, blood	65.64778
chr1:67686748..67686997	K562	HDAC2	bodily fluid, blood	124.95784
chr1:67686637..67687000	stomach	POLR2A	stomach	250.19110

chr1:67686363..67687053	HepG2	ZNF414	endocrine gland, exocrine gland, liver, epithelium	49.89714
chr1:67686638..67687108	K562	ZNF281	bodily fluid, blood	120.40944
chr1:67686428..67687019	GM12878	IKZF1	bodily fluid, blood	255.02806
chr1:67686697..67686990	GM12878	BHLHE40	bodily fluid, blood	163.62879
chr1:67686341..67686881	HEK293T	L3MBTL2	kidney, epithelium	127.00960
chr1:67686329..67687039	HepG2	ZNF343	endocrine gland, exocrine gland, liver, epithelium	41.11712
chr1:67686295..67686915	HepG2	ZSCAN31	endocrine gland, exocrine gland, liver, epithelium	42.92994
chr1:67686709..67687053	K562	GATAD2A	bodily fluid, blood	72.64946
chr1:67686486..67687046	HepG2	CREB1	endocrine gland, exocrine gland, liver, epithelium	72.89789
chr1:67686481..67687121	A549	JUN	lung	77.34535
chr1:67686701..67687131	K562	ZBTB2	bodily fluid, blood	80.65675
chr1:67686526..67687002	HepG2	HOXA3	endocrine gland, exocrine gland, liver, epithelium	16.80744
chr1:67686464..67686849	HepG2	AFF4	endocrine gland, exocrine gland, liver, epithelium	74.42097
chr1:67686715..67686943	K562	MTA3	bodily fluid, blood	116.76340
chr1:67686727..67687003	HCT116	ZBTB33	colon, large intestine, epithelium, intestine	13.68838
chr1:67686588..67686952	HEK293	ZNF280D	kidney, epithelium	42.24126
chr1:67686548..67687032	A549	EP300	lung	27.98109
chr1:67684758..67688906	K562	RBFOX2	bodily fluid, blood	227.50244
chr1:67684758..67688906	K562	RBFOX2	bodily fluid, blood	15.04667
chr1:67686240..67686780	HepG2	ERF	endocrine gland, exocrine gland, liver, epithelium	57.72228
chr1:67686608..67687028	A549	FOSL2	lung	48.57938
chr1:67686498..67686902	HepG2	SOX5	endocrine gland, exocrine gland, liver, epithelium	30.48822
chr1:67686325..67687005	K562	ZNF197	bodily fluid, blood	25.80327
chr1:67686590..67686933	upper lobe of left lung	POLR2A	lung	164.57992

chr1:67686440..67687030	HepG2	LCORL	endocrine gland, exocrine gland, liver, epithelium	47.58490
chr1:67686471..67686911	SK-N-SH	ZFP3	brain	67.16388
chr1:67685754..67686973	HepG2	TAF1	endocrine gland, exocrine gland, liver, epithelium	83.20124
chr1:67686566..67687016	HEK293	ZNF76	kidney, epithelium	64.59496
chr1:67686500..67687140	HepG2	SAFB2	endocrine gland, exocrine gland, liver, epithelium	31.72007
chr1:67686363..67686867	HepG2	HNF1B	endocrine gland, exocrine gland, liver, epithelium	49.98700
chr1:67686589..67687033	K562	MYC	bodily fluid, blood	60.76213
chr1:67686646..67687016	HEK293	KLF7	kidney, epithelium	48.95259
chr1:67686738..67687201	HEK293	PRDM6	kidney, epithelium	471.78243
chr1:67686560..67686910	K562	DDIT3	bodily fluid, blood	152.24296
chr1:67686641..67686862	K562	CEBPB	bodily fluid, blood	99.88742
chr1:67686426..67686926	GM12891	POLR2A	bodily fluid, blood	31.64597
chr1:67686431..67686761	K562	TAF7	bodily fluid, blood	45.45770
chr1:67686660..67686990	K562	TAF7	bodily fluid, blood	20.25773
chr1:67686647..67686939	liver	SP1	endocrine gland, exocrine gland, liver	119.65858
chr1:67686538..67687002	transverse colon	POLR2A	colon, large intestine, intestine	75.18850
chr1:67686744..67687208	transverse colon	POLR2A	colon, large intestine, intestine	16.65034
chr1:67684945..67687098	A549	JUN	lung	128.60052
chr1:67684945..67687098	A549	JUN	lung	90.03625
chr1:67684945..67687098	A549	JUN	lung	87.37065
chr1:67684945..67687098	A549	JUN	lung	343.99004
chr1:67686716..67686996	HEK293	ZBTB44	kidney, epithelium	192.88306
chr1:67686716..67687039	SK-N-SH	TCF12	brain	265.48475
chr1:67686430..67686794	esophagus squamous	CTCF	epithelium, esophagus	28.39389

chr1:67686439..67686955	OCI-LY7	CTCF	bodily fluid, blood	33.85473
chr1:67686729..67686969	upper lobe of left lung	EP300	lung	34.59782
chr1:67686752..67686976	HEK293	MZF1	kidney, epithelium	159.13021
chr1:67686406..67686870	K562	NR4A1	bodily fluid, blood	105.41146
chr1:67686743..67687083	K562	MTA2	bodily fluid, blood	53.45028
chr1:67686613..67687057	K562	DDX20	bodily fluid, blood	45.53660
chr1:67686668..67686971	GM12878	SKIL	bodily fluid, blood	130.23151
chr1:67686656..67687036	K562	TBP	bodily fluid, blood	39.22509
chr1:67686443..67686933	HepG2	ZNF609	endocrine gland, exocrine gland, liver, epithelium	48.14773
chr1:67686587..67686892	K562	AFF1	bodily fluid, blood	157.23640
chr1:67686690..67686940	K562	ATF1	bodily fluid, blood	66.75048
chr1:67686717..67687037	PFSK-1	REST	connective tissue, brain	36.36662
chr1:67686633..67687043	K562	MYC	bodily fluid, blood	52.25253
chr1:67686256..67686946	K562	ATF1	bodily fluid, blood	207.98865
chr1:67686458..67687148	K562	ATF1	bodily fluid, blood	96.76378
chr1:67686374..67687148	erythroblast	POLR2A	bone marrow, bone element	147.54355
chr1:67686534..67687127	K562	ATF3	bodily fluid, blood	213.51177
chr1:67686699..67687043	K562	ARID3A	bodily fluid, blood	69.53200
chr1:67686576..67686892	K562	GABPA	bodily fluid, blood	31.85683
chr1:67686562..67686932	liver	HNF4G	endocrine gland, exocrine gland, liver	51.81875
chr1:67686608..67686921	HepG2	ZGPAT	endocrine gland, exocrine gland, liver, epithelium	40.79895
chr1:67686728..67686998	IMR-90	MAFK	lung, connective tissue	43.85576
chr1:67686722..67687003	K562	BHLHE40	bodily fluid, blood	376.81086
chr1:67686623..67687003	K562	MYC	bodily fluid, blood	77.37643

chr1:67686441..67687041	HepG2	MYBL2	endocrine gland, exocrine gland, liver, epithelium	31.38127
chr1:67686712..67687088	H1	TBP	embryo	13.96770
chr1:67686591..67686976	liver	SP1	endocrine gland, exocrine gland, liver	205.88907
chr1:67686678..67686985	K562	MAX	bodily fluid, blood	59.95013
chr1:67686513..67686789	K562	ELF1	bodily fluid, blood	32.39083
chr1:67686547..67687011	A549	NR3C1	lung	48.45582
chr1:67686498..67686888	HepG2	LCOR	endocrine gland, exocrine gland, liver, epithelium	28.73549
chr1:67686303..67686783	HepG2	ZNF160	endocrine gland, exocrine gland, liver, epithelium	26.13082
chr1:67686507..67686987	HepG2	ZNF160	endocrine gland, exocrine gland, liver, epithelium	21.08171
chr1:67686630..67687014	neural cell	POLR2A		16.32213
chr1:67686141..67687076	body of pancreas	POLR2A	pancreas	758.57298
chr1:67686540..67687140	K562	HMG20A	bodily fluid, blood	28.88040
chr1:67686496..67687006	liver	TAF1	endocrine gland, exocrine gland, liver	62.24240
chr1:67686697..67687207	liver	TAF1	endocrine gland, exocrine gland, liver	14.31341
chr1:67686615..67687095	A549	JUNB	lung	34.21184
chr1:67686698..67686908	K562	NCOR1	bodily fluid, blood	134.50249
chr1:67686626..67687090	A549	JUNB	lung	70.08601
chr1:67684983..67687391	A549	JUN	lung	64.94883
chr1:67686393..67686857	A549	RAD21	lung	85.34381
chr1:67686700..67686980	K562	MAFF	bodily fluid, blood	98.45707
chr1:67686252..67686776	middle frontal area 46	CTCF	brain	26.07380
chr1:67686656..67686976	H1	BACH1	embryo	37.20240
chr1:67685726..67687073	A549	JUN	lung	106.75991
chr1:67686425..67686935	GM18505	POLR2A	bodily fluid, blood	63.19988

chr1:67686419..67686963	HepG2	IKZF5	endocrine gland, exocrine gland, liver, epithelium	41.62022
chr1:67686421..67686897	GM19099	POLR2A	bodily fluid, blood	19.93236
chr1:67686421..67686791	HepG2	ZNF331	endocrine gland, exocrine gland, liver, epithelium	28.48128
chr1:67686620..67686990	HepG2	ZNF331	endocrine gland, exocrine gland, liver, epithelium	14.47445
chr1:67684895..67687089	A549	JUN	lung	178.35952
chr1:67684895..67687089	A549	JUN	lung	144.59956
chr1:67684895..67687089	A549	JUN	lung	399.67267
chr1:67686554..67687054	HeLa-S3	TCF7L2	uterus, epithelium	84.53408
chr1:67686666..67686962	MCF 10A	MYC	mammary gland, exocrine gland, epithelium	62.61695
chr1:67686385..67686805	A549	ELF1	lung	26.39065
chr1:67686560..67686980	A549	CEBPB	lung	30.52716
chr1:67686697..67687187	liver	NR2F2	endocrine gland, exocrine gland, liver	20.22327
chr1:67686573..67687053	A549	NR3C1	lung	41.46072
chr1:67686650..67687004	HEK293	HIC1	kidney, epithelium	336.56131
chr1:67686565..67687178	HEK293	ZNF366	kidney, epithelium	380.99970
chr1:67686565..67687193	HEK293	ZNF366	kidney, epithelium	87.75173
chr1:67686684..67687003	HEK293	SP2	kidney, epithelium	182.00079
chr1:67686657..67687051	K562	TEAD4	bodily fluid, blood	255.04611
chr1:67686401..67686911	GM12891	POLR2A	bodily fluid, blood	51.16092
chr1:67685102..67687108	HEK293	ZBTB8A	kidney, epithelium	388.45497
chr1:67685102..67687108	HEK293	ZBTB8A	kidney, epithelium	96.41386
chr1:67685102..67687108	HEK293	ZBTB8A	kidney, epithelium	51.91008
chr1:67686525..67687116	HEK293	WT1	kidney, epithelium	893.99548
chr1:67686676..67687046	HEK293	BCL11B	kidney, epithelium	77.28406

chr1:67686411..67686901	HEK293	ZFP37	kidney, epithelium	127.63163
chr1:67686350..67686880	HepG2	ZNF891	endocrine gland, exocrine gland, liver, epithelium	26.42357
chr1:67686466..67687210	K562	ZNF584	bodily fluid, blood	43.14510
chr1:67686436..67687128	transverse colon	POLR2A	colon, large intestine, intestine	82.22318
chr1:67686670..67686940	HepG2	JUND	endocrine gland, exocrine gland, liver, epithelium	63.56445
chr1:67686585..67686915	K562	E2F4	bodily fluid, blood	18.16855
chr1:67686676..67686925	HepG2	ARID5B	endocrine gland, exocrine gland, liver, epithelium	73.24455
chr1:67686487..67687037	A549	BCL3	lung	28.50019
chr1:67686690..67687240	A549	BCL3	lung	15.99035
chr1:67686620..67687056	HeLa-S3	GTF2F1	uterus, epithelium	52.97671
chr1:67686624..67686844	HepG2	CEBPB	endocrine gland, exocrine gland, liver, epithelium	58.13706
chr1:67686155..67686859	HepG2	UBTF	endocrine gland, exocrine gland, liver, epithelium	96.66931
chr1:67686378..67686958	HepG2	KMT2A	endocrine gland, exocrine gland, liver, epithelium	51.86004
chr1:67686556..67686960	HEK293	ZNF610	kidney, epithelium	54.23728
chr1:67686487..67686871	HEK293	ZNF10	kidney, epithelium	121.26045
chr1:67686688..67687072	HEK293	ZNF10	kidney, epithelium	98.57447
chr1:67686704..67687128	K562	ARNT	bodily fluid, blood	82.13466
chr1:67686625..67687055	HCT116	CBX3	colon, large intestine, epithelium, intestine	35.41860
chr1:67686610..67686880	K562	CEBPB	bodily fluid, blood	92.23882
chr1:67686490..67686870	K562	ETS1	bodily fluid, blood	37.26702
chr1:67686556..67687040	K562	POLR2A	bodily fluid, blood	28.54142
chr1:67686630..67686853	gastroesophageal sphincter	POLR2A	musculature of body, stomach	157.73641
chr1:67686465..67686889	K562	KLF1	bodily fluid, blood	74.94400
chr1:67686451..67686761	K562	HDAC2	bodily fluid, blood	27.08930

chr1:67686751..67686858	MCF-7	REST	mammary gland, exocrine gland, epithelium	80.21600
chr1:67686677..67687087	K562	GATA2	bodily fluid, blood	74.35757
chr1:67686683..67687052	K562	TAF9B	bodily fluid, blood	470.29837
chr1:67686717..67687133	gastroesophageal sphincter	POLR2A	musculature of body, stomach	5.78781
chr1:67686751..67687001	K562	GATA2	bodily fluid, blood	85.88210
chr1:67686406..67686846	GM12878	LARP7	bodily fluid, blood	91.16774
chr1:67686415..67687219	HepG2	ASH2L	endocrine gland, exocrine gland, liver, epithelium	18.26394
chr1:67686308..67686848	HepG2	HIVEP1	endocrine gland, exocrine gland, liver, epithelium	70.07940
chr1:67686348..67687032	HepG2	ZNF335	endocrine gland, exocrine gland, liver, epithelium	41.02207
chr1:67686631..67686961	K562	TAF7	bodily fluid, blood	20.04262
chr1:67686563..67687047	liver	REST	endocrine gland, exocrine gland, liver	31.29873
chr1:67686438..67686814	sigmoid colon	POLR2A	colon, large intestine, intestine	42.13762
chr1:67686622..67686998	HepG2	ARID3A	endocrine gland, exocrine gland, liver, epithelium	28.80926
chr1:67685917..67686761	neural progenitor cell	EZH2		23.85882
chr1:67686675..67687099	HEK293	EGR2	kidney, epithelium	33.31960
chr1:67686656..67687096	HEK293	ZNF18	kidney, epithelium	60.11376
chr1:67686532..67687002	A549	NR3C1	lung	30.60647
chr1:67686719..67686931	HEK293	ZIC2	kidney, epithelium	110.26090
chr1:67686662..67687002	SK-N-SH	SIN3A	brain	41.64937
chr1:67686616..67687032	A549	RAD21	lung	40.17416
chr1:67686556..67687040	H1	TAF1	embryo	6.99874
chr1:67686571..67687027	A549	RAD21	lung	46.31326
chr1:67686588..67687032	HepG2	HES4	endocrine gland, exocrine gland, liver, epithelium	17.82838
chr1:67686488..67686968	A549	NR3C1	lung	15.27590

chr1:67686684..67687208	liver	GABPA	endocrine gland, exocrine gland, liver	8.48035
chr1:67686639..67686829	endothelial cell of umbilical vein	POLR2A	blood vessel, vein, placenta, extraembryonic component, vasculature, epithelium	29.65989
chr1:67686613..67686974	endothelial cell of umbilical vein	POLR2A	blood vessel, vein, placenta, extraembryonic component, vasculature, epithelium	86.41999
chr1:67686523..67686993	K562	POLR2A	bodily fluid, blood	39.94281
chr1:67686422..67686972	HepG2	PAXIP1	endocrine gland, exocrine gland, liver, epithelium	77.00741
chr1:67686637..67686875	HepG2	THAP11	endocrine gland, exocrine gland, liver, epithelium	50.36558
chr1:67686730..67687410	A549	JUN	lung	22.01419
chr1:67686306..67686783	K562	RBBP5	bodily fluid, blood	30.42325
chr1:67686716..67687040	HeLa-S3	RFX5	uterus, epithelium	39.72867
chr1:67686738..67686968	HeLa-S3	RAD21	uterus, epithelium	52.22647
chr1:67686515..67686811	K562	THAP12	bodily fluid, blood	65.29869
chr1:67686741..67686983	endothelial cell of umbilical vein	FOS	blood vessel, vein, placenta, extraembryonic component, vasculature, epithelium	227.24323
chr1:67686444..67687140	HepG2	YEATS4	endocrine gland, exocrine gland, liver, epithelium	38.81503
chr1:67686212..67686908	HepG2	YEATS4	endocrine gland, exocrine gland, liver, epithelium	67.89150
chr1:67686374..67686804	upper lobe of left lung	CTCF	lung	26.86476
chr1:67686355..67686815	heart left ventricle	CTCF	heart	27.18924
chr1:67686218..67686782	HepG2	SRY	endocrine gland, exocrine gland, liver, epithelium	62.87901
chr1:67686552..67686932	HepG2	FOXA2	endocrine gland, exocrine gland, liver, epithelium	45.66629
chr1:67686450..67687074	HepG2	ZNF430	endocrine gland, exocrine gland, liver, epithelium	54.86432
chr1:67686405..67687188	HEK293	ZNF394	kidney, epithelium	3604.69297
chr1:67684684..67686928	HEK293	ZBTB26	kidney, epithelium	1103.71494
chr1:67684684..67686928	HEK293	ZBTB26	kidney, epithelium	497.53594
chr1:67686487..67686891	upper lobe of left lung	POLR2A	lung	43.85849
chr1:67686725..67687069	SK-N-SH	CREB5	brain	55.55731

chr1:67686545..67686975	HepG2	FOXP4	endocrine gland, exocrine gland, liver, epithelium	34.00747
chr1:67686723..67687093	SK-N-SH	RXRA	brain	40.93245
chr1:67686740..67686862	SK-N-SH	MAX	brain	70.83378
chr1:67686588..67687068	HepG2	TOE1	endocrine gland, exocrine gland, liver, epithelium	31.27591
chr1:67686622..67686966	SK-N-SH	ELF1	brain	21.52441
chr1:67686502..67686886	GM12878	TBP	bodily fluid, blood	22.16876
chr1:67686322..67686778	middle frontal area 46	CTCF	brain	19.12829
chr1:67686690..67686986	K562	JUN	bodily fluid, blood	66.23893
chr1:67686687..67686911	HepG2	RAD21	endocrine gland, exocrine gland, liver, epithelium	13.28132
chr1:67686598..67687038	K562	JUN	bodily fluid, blood	33.98141
chr1:67686666..67687036	K562	MAZ	bodily fluid, blood	29.69033
chr1:67686667..67686858	liver	ATF3	endocrine gland, exocrine gland, liver	119.25108
chr1:67686659..67686978	K562	ZNF316	bodily fluid, blood	416.50060
chr1:67686433..67686877	A549	SMC3	lung	63.89621
chr1:67686612..67686976	HEK293	HOXD13	kidney, epithelium	22.43009
chr1:67686621..67686991	upper lobe of left lung	POLR2A	lung	70.05667
chr1:67686231..67686801	HepG2	ZNF574	endocrine gland, exocrine gland, liver, epithelium	41.27158
chr1:67686404..67686814	K562	UBTF	bodily fluid, blood	31.82069
chr1:67686607..67687017	K562	UBTF	bodily fluid, blood	9.52688
chr1:67686581..67687031	K562	NFATC3	bodily fluid, blood	88.32549
chr1:67686696..67686992	K562	JUN	bodily fluid, blood	41.11505
chr1:67686645..67686983	GM12878	IKZF2	bodily fluid, blood	258.13304
chr1:67686478..67686908	HEK293	REST	kidney, epithelium	55.57068
chr1:67686428..67686899	BLaER1	CEBPA	bone marrow, bone element	4.96920

chr1:67686659..67686963	MCF-7	MYC	mammary gland, exocrine gland, epithelium	41.86216
chr1:67686624..67687084	K562	ZNF639	bodily fluid, blood	57.40429
chr1:67686511..67687027	GM12878	ATF7	bodily fluid, blood	58.01927
chr1:67686731..67687021	T47D	GATA3	mammary gland, exocrine gland, epithelium	26.94289
chr1:67686616..67687000	HeLa-S3	MXI1	uterus, epithelium	35.55668
chr1:67686736..67686972	K562	JUN	bodily fluid, blood	23.66523
chr1:67686321..67686971	K562	KDM5B	bodily fluid, blood	33.75221
chr1:67686686..67686898	GM12878	RUNX3	bodily fluid, blood	173.93367
chr1:67686615..67686959	K562	IRF2	bodily fluid, blood	29.46638
chr1:67686649..67687000	liver	RAD21	endocrine gland, exocrine gland, liver	214.21678
chr1:67686341..67686941	HepG2	HDAC1	endocrine gland, exocrine gland, liver, epithelium	38.21736
chr1:67686713..67686896	K562	E4F1	bodily fluid, blood	120.36129
chr1:67686699..67686854	transverse colon	POLR2A	colon, large intestine, intestine	100.86870
chr1:67686474..67687214	K562	BACH1	bodily fluid, blood	89.25806
chr1:67686640..67687003	sigmoid colon	POLR2A	colon, large intestine, intestine	159.16319
chr1:67686675..67687091	K562	ZNF184	bodily fluid, blood	85.52086
chr1:67686484..67686904	HepG2	DRAP1	endocrine gland, exocrine gland, liver, epithelium	34.27878
chr1:67686274..67686794	HepG2	MXD3	endocrine gland, exocrine gland, liver, epithelium	59.07006
chr1:67686445..67686989	HepG2	SPEN	endocrine gland, exocrine gland, liver, epithelium	26.19269
chr1:67686369..67686765	esophagus squamous	CTCF	epithelium, esophagus	24.93575
chr1:67686663..67686947	SK-N-SH	GLIS3	brain	45.65123
chr1:67686638..67686974	HepG2	ZNF644	endocrine gland, exocrine gland, liver, epithelium	25.55830
chr1:67686630..67686901	K562	CEBPG	bodily fluid, blood	301.15194
chr1:67686691..67687251	K562	CEBPG	bodily fluid, blood	24.90285

chr1:67686312..67686916	A549	JUN	lung	66.50776
chr1:67686732..67686931	A549	JUN	lung	55.43304
chr1:67686512..67687042	esophagus muscularis	POLR2A	esophagus, musculature of body	620.64173
chr1:67686699..67687043	stomach	POLR2A	stomach	41.40161
chr1:67684917..67687504	K562	POLR2A	bodily fluid, blood	1620.52008
chr1:67684917..67687504	K562	POLR2A	bodily fluid, blood	144.86697
chr1:67686282..67686818	HepG2	ZNF317	endocrine gland, exocrine gland, liver, epithelium	68.95137
chr1:67686735..67687095	K562	ARNT	bodily fluid, blood	40.92761
chr1:67686557..67687138	K562	CBFA2T3	bodily fluid, blood	1501.88787
chr1:67686222..67687081	HeLa-S3	POLR2A	uterus, epithelium	175.66472
chr1:67686633..67687183	K562	TOE1	bodily fluid, blood	90.49991
chr1:67686673..67686990	K562	MNT	bodily fluid, blood	181.10440
chr1:67686590..67687054	A549	JUNB	lung	34.91288
chr1:67686401..67687025	HepG2	MAZ	endocrine gland, exocrine gland, liver, epithelium	22.88085
chr1:67686650..67687020	HeLa-S3	RCOR1	uterus, epithelium	57.63311
chr1:67686152..67687016	K562	ZBTB11	bodily fluid, blood	64.03407
chr1:67686519..67686877	esophagus squamous	POLR2A	epithelium, esophagus	247.05369
chr1:67686545..67686965	HepG2	GTF2F1	endocrine gland, exocrine gland, liver, epithelium	28.12486
chr1:67686293..67686973	HepG2	ZNF350	endocrine gland, exocrine gland, liver, epithelium	40.45899
chr1:67686719..67686939	suprapubic skin	POLR2A	skin of body	12.01768
chr1:67686662..67687068	K562	RNF2	bodily fluid, blood	245.64252
chr1:67686388..67687039	right lobe of liver	POLR2A	endocrine gland, exocrine gland, liver	107.87683
chr1:67686388..67687039	right lobe of liver	POLR2A	endocrine gland, exocrine gland, liver	232.39354
chr1:67686139..67686889	HepG2	ZNF501	endocrine gland, exocrine gland, liver, epithelium	79.08128

chr1:67686636..67687016	HepG2	MNT	endocrine gland, exocrine gland, liver, epithelium	40.02235
chr1:67684873..67686892	spleen	POLR2A	spleen, immune organ	120.41549
chr1:67686412..67687120	HEK293	ZEB2	kidney, epithelium	380.24660
chr1:67686621..67686891	K562	ZSCAN32	bodily fluid, blood	43.87545
chr1:67686436..67687016	HepG2	SOX6	endocrine gland, exocrine gland, liver, epithelium	119.75690
chr1:67686651..67686850	HEK293	BCL11A	kidney, epithelium	133.14853
chr1:67684873..67686892	spleen	POLR2A	spleen, immune organ	7278.62354
chr1:67684873..67686892	spleen	POLR2A	spleen, immune organ	301.73167
chr1:67686604..67687060	GM12878	ELF1	bodily fluid, blood	86.80166
chr1:67686456..67686972	HepG2	ZNF788P	endocrine gland, exocrine gland, liver, epithelium	52.35249
chr1:67686696..67686835	HEK293	ZFP69B	kidney, epithelium	73.38235
chr1:67686666..67687116	K562	BRD9	bodily fluid, blood	54.40383
chr1:67686608..67686874	body of pancreas	POLR2A	pancreas	237.83720
chr1:67686635..67686964	spleen	POLR2A	spleen, immune organ	110.60858
chr1:67686577..67686967	MCF-7	ELF1	mammary gland, exocrine gland, epithelium	41.18455
chr1:67686463..67686999	A549	BCL3	lung	14.73248
chr1:67686501..67687005	A549	EP300	lung	33.01589
chr1:67686651..67687031	K562	ZNF7	bodily fluid, blood	35.83238
chr1:67686449..67686829	K562	ZNF7	bodily fluid, blood	43.86103
chr1:67684816..67686979	HepG2	ZNF687	endocrine gland, exocrine gland, liver, epithelium	423.88706
chr1:67686671..67687051	HEK293	GLIS1	kidney, epithelium	224.40352
chr1:67686125..67687142	HEK293	ZBTB17	kidney, epithelium	290.85109
chr1:67686619..67686995	HepG2	TEAD1	endocrine gland, exocrine gland, liver, epithelium	35.45547
chr1:67686275..67686805	MCF-7	SIN3A	mammary gland, exocrine gland, epithelium	51.99326

chr1:67684889..67687202	K562	POLR2G	bodily fluid, blood	501.72784
chr1:67684889..67687202	K562	POLR2G	bodily fluid, blood	275.17169
chr1:67684889..67687202	K562	POLR2G	bodily fluid, blood	60.59719
chr1:67686157..67686833	HepG2	KDM5B	endocrine gland, exocrine gland, liver, epithelium	84.58270
chr1:67686480..67686945	esophagus muscularis	POLR2A	esophagus, musculature of body	109.72013
chr1:67686341..67686781	K562	FOXK1	bodily fluid, blood	54.19824
chr1:67686379..67686863	HepG2	ZNF614	endocrine gland, exocrine gland, liver, epithelium	41.41958
chr1:67686732..67687088	K562	TRIM25	bodily fluid, blood	47.25289
chr1:67686361..67687031	K562	VEZF1	bodily fluid, blood	72.03486
chr1:67686592..67687052	A549	NR3C1	lung	22.59826
chr1:67686734..67687144	K562	FOXM1	bodily fluid, blood	59.24595
chr1:67686473..67687089	K562	MNT	bodily fluid, blood	119.89084
chr1:67686706..67687042	K562	ZMIZ1	bodily fluid, blood	41.36746
chr1:67686255..67686831	A549	BCL3	lung	11.80778
chr1:67686460..67687238	K562	MLLT1	bodily fluid, blood	33.60199
chr1:67686748..67686988	SK-N-SH	USF1	brain	30.85490
chr1:67686599..67687095	A549	NR3C1	lung	37.30036
chr1:67685699..67687162	MCF 10A	POLR2A	mammary gland, exocrine gland, epithelium	73.50408
chr1:67686509..67686899	esophagus squamous	POLR2A	epithelium, esophagus	73.53585
chr1:67686488..67687084	K562	CHD4	bodily fluid, blood	13.14469
chr1:67686275..67686871	K562	CHD4	bodily fluid, blood	12.78436
chr1:67686673..67687113	GM12878	RAD51	bodily fluid, blood	37.31191
chr1:67686677..67686927	BLaER1	CEBPA	bone marrow, bone element	3.98427
chr1:67686441..67686911	tibial nerve	POLR2A	nerve, limb	28.65196

chr1:67686680..67686960	stomach	EP300	stomach	21.57877
chr1:67686559..67687189	A549	JUN	lung	21.42678
chr1:67686698..67687042	K562	KLF16	bodily fluid, blood	90.10136
chr1:67686486..67686966	K562	ID3	bodily fluid, blood	83.36895
chr1:67686690..67687170	K562	ID3	bodily fluid, blood	29.98710
chr1:67686361..67687021	HepG2	SFPQ	endocrine gland, exocrine gland, liver, epithelium	28.51928
chr1:67686706..67687230	HepG2	ZNF446	endocrine gland, exocrine gland, liver, epithelium	38.77720
chr1:67685614..67687036	HEK293	PATZ1	kidney, epithelium	129.17283
chr1:67686538..67687048	prostate gland	POLR2A	prostate gland	42.10915
chr1:67686323..67686803	K562	CREB1	bodily fluid, blood	50.32758
chr1:67686577..67686917	HepG2	HMG20B	endocrine gland, exocrine gland, liver, epithelium	36.34763
chr1:67686632..67687072	HEK293	ZNF561	kidney, epithelium	83.39274
chr1:67686676..67687080	Peyer's patch	POLR2A	intestine, lymphoid tissue	41.42343
chr1:67686168..67686884	HepG2	ZNF337	endocrine gland, exocrine gland, liver, epithelium	45.99906
chr1:67686631..67686883	HepG2	MAX	endocrine gland, exocrine gland, liver, epithelium	72.16473
chr1:67686241..67686797	HepG2	EGR1	endocrine gland, exocrine gland, liver, epithelium	67.14920
chr1:67686592..67686936	HEK293	ZSCAN18	kidney, epithelium	32.44231
chr1:67686708..67686798	HepG2	CEBPD	endocrine gland, exocrine gland, liver, epithelium	49.48718
chr1:67686575..67686965	HepG2	ZNF697	endocrine gland, exocrine gland, liver, epithelium	24.24967
chr1:67686500..67687070	A549	TCF12	lung	23.09987
chr1:67686661..67686876	HepG2	ZNF48	endocrine gland, exocrine gland, liver, epithelium	38.50687
chr1:67686330..67687008	gastrocnemius medialis	POLR2A	musculature of body, limb	38.16241
chr1:67686484..67687044	HepG2	KAT8	endocrine gland, exocrine gland, liver, epithelium	41.16624
chr1:67685855..67686941	HepG2	GABPB1	endocrine gland, exocrine gland, liver, epithelium	135.84571

chr1:67685855..67686941	HepG2	GABPB1	endocrine gland, exocrine gland, liver, epithelium	51.55000
chr1:67686257..67686801	HepG2	ZNF786	endocrine gland, exocrine gland, liver, epithelium	46.04596
chr1:67686633..67686933	K562	ATF4	bodily fluid, blood	165.57799
chr1:67686672..67686992	HEK293	ZBTB1	kidney, epithelium	41.53130
chr1:67686412..67686882	HepG2	PITX1	endocrine gland, exocrine gland, liver, epithelium	62.44431
chr1:67686667..67686986	HEK293	ZNF24	kidney, epithelium	167.91473
chr1:67686675..67687005	HEK293	ZBTB12	kidney, epithelium	84.61456
chr1:67686715..67686975	upper lobe of left lung	EP300	lung	79.42995
chr1:67686499..67687059	K562	CXXC5	bodily fluid, blood	41.94925
chr1:67686570..67686980	heart left ventricle	CTCF	heart	68.59817
chr1:67686495..67686979	K562	ZNF24	bodily fluid, blood	82.88660

Table S3. Chip-seq data obtained for rs225014 by Regulome DB.

**Peak location**	**Biosample**	**Targets**	**Organ**	**Value**
chr14:80203035..80203279	K562	CTCF	bodily fluid, blood	15.49222
chr14:80203047..80203247	A549	CTCF	lung	23.21889
chr14:80203024..80203250	GM12873	CTCF	bodily fluid, blood	242.46257
chr14:80203008..80203318	adrenal gland	CTCF	endocrine gland, adrenal gland	39.61489
chr14:80202958..80203318	keratinocyte	CTCF	skin of body, epithelium	57.31055
chr14:80202995..80203265	HepG2	SMC3	endocrine gland, exocrine gland, liver, epithelium	20.67121
chr14:80203077..80203617	LNCAP	CTCF	prostate gland	61.80729
chr14:80203017..80203287	GM12878	SMC3	bodily fluid, blood	46.86747
chr14:80203050..80203240	GM12878	RAD21	bodily fluid, blood	39.04798
chr14:80202999..80203269	mammary epithelial cell	CTCF	mammary gland, exocrine gland, epithelium	38.24696

chr14:80203026..80203242	HCT116	CTCF	colon, large intestine, epithelium, intestine	74.37176
chr14:80202606..80203688	C4-2B	CTCF	prostate gland	474.39927
chr14:80202969..80203285	fibroblast of lung	CTCF	lung, connective tissue	42.41029
chr14:80202923..80203339	A549	RAD21	lung	27.09549
chr14:80203054..80203278	Ishikawa	CTCF	uterus	13.89399
chr14:80202941..80203310	SU-DHL-6	CTCF		145.90310
chr14:80203044..80203604	SU-DHL-6	CTCF		28.82287
chr14:80202864..80203395	bipolar neuron	CTCF	spinal cord, eye, brain, ear	483.09580
chr14:80202901..80203405	HCT116	CTCF	colon, large intestine, epithelium, intestine	45.13077
chr14:80202955..80203251	AG04450	CTCF	lung, connective tissue	32.04106
chr14:80202917..80203406	OCI-LY7	CTCF	bodily fluid, blood	56.89617
chr14:80202863..80203393	PC-9	CTCF	lung	40.47213
chr14:80203035..80203285	SK-N-SH	RAD21	brain	78.88840
chr14:80203039..80203241	WTC11	CTCF	skin of body, connective tissue	49.68514
chr14:80202956..80203660	activated CD4-positive, alpha- [illegible]	CTCF	bodily fluid, blood	41.71130
chr14:80202946..80203316	MCF-7	TRIM22	mammary gland, exocrine gland, epithelium	44.14592
chr14:80202911..80203347	upper lobe of right lung	CTCF	lung	32.03270
chr14:80203021..80203247	GM12864	CTCF	bodily fluid, blood	193.26580
chr14:80203029..80203239	spleen	CTCF	spleen, immune organ	16.66594
chr14:80203032..80203256	Ishikawa	RAD21	uterus	75.54798
chr14:80202930..80203274	astrocyte of the cerebellum	CTCF	brain	25.37191
chr14:80203044..80203240	endothelial cell of umbilical vein	CTCF	blood vessel, vein, placenta, extraembryonic component, vasculature, epithelium	52.96356
chr14:80202884..80203264	tibial nerve	CTCF	nerve, limb	12.93521
chr14:80202882..80203372	CD14-positive monocyte	CTCF	bodily fluid, blood	25.76360

chr14:80203082..80203572	CD14-positive monocyte	CTCF	bodily fluid, blood	7.01082
chr14:80202885..80203429	HCT116	CTCF	colon, large intestine, epithelium, intestine	55.57708
chr14:80202899..80203396	epithelial cell of prostate	CTCF	epithelium, prostate gland	89.22117
chr14:80202922..80203366	Caco-2	CTCF	colon, large intestine, intestine	35.00530
chr14:80202992..80203252	astrocyte of the spinal cord	CTCF	spinal cord	26.29534
chr14:80202961..80203317	GM12878	CTCF	bodily fluid, blood	88.59373
chr14:80202949..80203307	A673	CTCF	musculature of body	204.65608
chr14:80203030..80203246	HCT116	CTCF	colon, large intestine, epithelium, intestine	84.58237
chr14:80202990..80203297	Loucy	CTCF	bodily fluid, blood	176.21607
chr14:80202975..80203295	fibroblast of villous	CTCF	placenta, connective tissue, extraembryonic component	55.99822
chr14:80202898..80203398	HCT116	CTCF	colon, large intestine, epithelium, intestine	34.37698
chr14:80203026..80203242	GM12878	CTCF	bodily fluid, blood	135.53598
chr14:80202914..80203350	MCF-7	MAX	mammary gland, exocrine gland, epithelium	32.52754
chr14:80203029..80203245	fibroblast of the aortic adventitia	CTCF	arterial blood vessel, blood vessel, vasculature, connective tissue	35.40417
chr14:80202897..80203393	HCT116	CTCF	colon, large intestine, epithelium, intestine	45.08333
chr14:80202951..80203320	GM23338	CTCF	skin of body	280.12755
chr14:80202884..80203384	A549	CTCF	lung	73.93290
chr14:80202967..80203337	myotube	CTCF	musculature of body	17.35794
chr14:80203031..80203275	HL-60	CTCF	bodily fluid, blood	25.60671
chr14:80203006..80203276	K562	CTCF	bodily fluid, blood	27.08997
chr14:80203025..80203273	placenta	CTCF	placenta, extraembryonic component	118.88115
chr14:80203029..80203241	GM23338	CTCF	skin of body	253.80912
chr14:80202942..80203286	hepatocyte	CTCF	endocrine gland, exocrine gland, liver, epithelium	27.35254
chr14:80202993..80203260	HCT116	CTCF	colon, large intestine, epithelium, intestine	164.06622

chr14:80203012..80203256	IMR-90	CTCF	lung, connective tissue	12.57267
chr14:80202987..80203277	GM12878	ZNF143	bodily fluid, blood	46.75788
chr14:80202981..80203291	BJ	CTCF	skin of body, connective tissue, penis	45.80329
chr14:80202918..80203282	MCF 10A	CTCF	mammary gland, exocrine gland, epithelium	14.14850
chr14:80203001..80203251	HeLa-S3	CTCF	uterus, epithelium	49.48615
chr14:80203056..80203248	HCT116	CTCF	colon, large intestine, epithelium, intestine	61.75288
chr14:80203033..80203283	A549	RAD21	lung	40.07161
chr14:80202823..80203483	22Rv1	CTCF	prostate gland	496.12519
chr14:80202958..80203337	HFFc6	CTCF	connective tissue, penis	184.53231
chr14:80202958..80203337	HFFc6	CTCF	connective tissue, penis	47.95689
chr14:80202767..80203517	22Rv1	CTCF	prostate gland	254.66984
chr14:80202960..80203312	foreskin keratinocyte	CTCF	skin of body, epithelium, penis	216.42394
chr14:80202871..80203375	nephron progenitor cell	CTCF	kidney	48.58170
chr14:80202905..80203325	parathyroid adenoma	CTCF	paraythroid gland	48.26443
chr14:80202955..80203399	suprapubic skin	CTCF	skin of body	24.43636
chr14:80202865..80203395	HCT116	CTCF	colon, large intestine, epithelium, intestine	61.86890
chr14:80203014..80203243	HepG2	CTCF	endocrine gland, exocrine gland, liver, epithelium	235.43030
chr14:80202965..80203311	foreskin keratinocyte	CTCF	skin of body, epithelium, penis	280.46133
chr14:80202951..80203291	Peyer's patch	CTCF	intestine, lymphoid tissue	47.58257
chr14:80202996..80203245	type B pancreatic cell	CTCF	pancreas, endocrine gland, epithelium	98.56340
chr14:80202935..80203411	A549	CTCF	lung	56.82417
chr14:80202989..80203279	testis	CTCF	gonad, testis	36.17762
chr14:80202979..80203303	keratinocyte	CTCF	skin of body, epithelium	53.63132
chr14:80202982..80203258	choroid plexus epithelial cell	CTCF	vasculature, brain, epithelium	53.61918

chr14:80203033..80203244	HEK293	CTCF	kidney, epithelium	169.72167
chr14:80202908..80203334	endodermal cell	CTCF	embryo	459.57128
chr14:80202945..80203333	chondrocyte	CTCF	connective tissue	112.54984
chr14:80202946..80203386	heart left ventricle	CTCF	heart	23.86665
chr14:80203074..80203678	GM23338	CTCF	skin of body	31.47809
chr14:80202950..80203320	thyroid gland	CTCF	thyroid gland, endocrine gland	39.32753
chr14:80202926..80203396	neural crest cell	CTCF	embryo	75.78926
chr14:80202796..80203366	mesothelial cell of epicardium	CTCF	heart, pericardium, epithelium	23.47658
chr14:80202827..80203467	excitatory neuron	CTCF		92.43628
chr14:80202944..80203308	K562	RAD21	bodily fluid, blood	62.32609
chr14:80202943..80203303	MCF-7	NR2F2	mammary gland, exocrine gland, epithelium	19.53476
chr14:80202738..80203442	activated CD4-positive, alpha- [illegible]	CTCF	bodily fluid, blood	67.25967
chr14:80202606..80203688	C4-2B	CTCF	prostate gland	1208.20779
chr14:80203073..80203309	IMR-90	RAD21	lung, connective tissue	18.20778
chr14:80203006..80203262	AG10803	CTCF	skin of body, connective tissue	43.43031
chr14:80202886..80203366	testis	CTCF	gonad, testis	26.78452
chr14:80202917..80203373	lower lobe of right lung	CTCF	lung	22.59178
chr14:80202865..80203524	RWPE2	CTCF	epithelium, prostate gland	308.87012
chr14:80202865..80203524	RWPE2	CTCF	epithelium, prostate gland	156.10939
chr14:80202902..80203358	heart right ventricle	CTCF	heart	47.01809
chr14:80202956..80203333	foreskin keratinocyte	CTCF	skin of body, epithelium, penis	348.02447
chr14:80202991..80203287	kidney epithelial cell	CTCF	kidney, epithelium	33.85328
chr14:80203029..80203269	K562	CTCF	bodily fluid, blood	25.96447
chr14:80202913..80203383	heart right ventricle	CTCF	heart	31.43535

chr14:80202853..80203433	neural progenitor cell	CTCF		51.21250
chr14:80203012..80203262	SK-N-SH	CTCF	brain	47.78921
chr14:80203033..80203297	GM12878	RAD21	bodily fluid, blood	33.65410
chr14:80202899..80203396	epithelial cell of prostate	CTCF	epithelium, prostate gland	243.59108
chr14:80202976..80203305	neural progenitor cell	CTCF		266.10539
chr14:80203022..80203298	NB4	MAX	bone marrow, bone element	43.45834
chr14:80203018..80203258	H1	NANOG	embryo	20.52805
chr14:80202953..80203337	SK-N-SH	CTCF	brain	215.16609
chr14:80202953..80203337	SK-N-SH	CTCF	brain	11.97823
chr14:80202931..80203307	HFF-Myc	CTCF	connective tissue, penis	106.07201
chr14:80203003..80203243	SK-N-SH	SMC3	brain	22.50725
chr14:80202940..80203276	H1	YY1	embryo	32.24289
chr14:80202895..80203425	HCT116	CTCF	colon, large intestine, epithelium, intestine	29.26264
chr14:80202851..80203447	KMS-11	CTCF	bone element	42.77581
chr14:80203054..80203650	KMS-11	CTCF	bone element	15.50134
chr14:80202999..80203249	NB4	CTCF	bone marrow, bone element	60.62929
chr14:80202992..80203288	fibroblast of dermis	CTCF	skin of body, connective tissue	46.17759
chr14:80203030..80203246	H1	CTCF	embryo	148.72807
chr14:80203020..80203284	fibroblast of mammary gland	CTCF	mammary gland, exocrine gland, connective tissue	101.95166
chr14:80202948..80203292	astrocyte	CTCF	spinal cord, brain	14.86224
chr14:80203017..80203261	A549	CTCF	lung	42.68283
chr14:80203006..80203313	HCT116	CTCF	colon, large intestine, epithelium, intestine	110.11648
chr14:80203004..80203288	HCT116	CTCF	colon, large intestine, epithelium, intestine	68.45741
chr14:80202948..80203312	cardiac muscle cell	CTCF	heart, musculature of body	51.08576

chr14:80203001..80203291	H1	ZNF143	embryo	46.80084
chr14:80203019..80203259	T47D	CTCF	mammary gland, exocrine gland, epithelium	19.33269
chr14:80202978..80203256	OCI-LY1	CTCF	bone marrow, bone element	161.67336
chr14:80203000..80203264	mammary epithelial cell	CTCF	mammary gland, exocrine gland, epithelium	71.84649
chr14:80202999..80203289	skeletal muscle myoblast	CTCF	musculature of body	26.04180
chr14:80203013..80203269	HepG2	RAD21	endocrine gland, exocrine gland, liver, epithelium	45.84572
chr14:80202995..80203271	AG09319	CTCF	mouth, connective tissue	60.96157
chr14:80203010..80203266	retinal pigment epithelial cell	CTCF	epithelium, eye	102.98713
chr14:80202979..80203239	fibroblast of lung	CTCF	lung, connective tissue	59.44724
chr14:80202923..80203343	MM.1S	CTCF	bodily fluid, blood	44.89553
chr14:80202911..80203401	A549	CTCF	lung	29.08617
chr14:80202908..80203390	endodermal cell	CTCF	embryo	51.10938
chr14:80202873..80203477	GM23338	CTCF	skin of body	71.53468
chr14:80202891..80203351	A549	CTCF	lung	55.18914
chr14:80203090..80203550	A549	CTCF	lung	15.12060
chr14:80203050..80203249	GM12872	CTCF	bodily fluid, blood	271.86676
chr14:80203149..80203519	thyroid gland	CTCF	thyroid gland, endocrine gland	9.68468
chr14:80202802..80203432	VCaP	CTCF	epithelium, prostate gland	70.96735
chr14:80203045..80203264	endothelial cell	CTCF	epithelium	85.85815
chr14:80202900..80203356	osteocyte	CTCF	connective tissue, bone element	80.80224
chr14:80202885..80203361	A549	CTCF	lung	65.30037
chr14:80203081..80203249	H1	MAX	embryo	83.26436
chr14:80202915..80203279	cardiac muscle cell	CTCF	heart, musculature of body	22.17564
chr14:80202980..80203274	LNCAP	CTCF	prostate gland	235.42098

chr14:80202997..80203567	mesothelial cell of epicardium	CTCF	heart, pericardium, epithelium	9.30457
chr14:80202887..80203343	H9	CTCF	embryo	516.70207
chr14:80202864..80203328	A549	CTCF	lung	43.37608
chr14:80202823..80203501	22Rv1	CTCF	prostate gland	178.49892
chr14:80202951..80203347	transverse colon	CTCF	colon, large intestine, intestine	33.38058
chr14:80202767..80203517	22Rv1	CTCF	prostate gland	461.01652
chr14:80202968..80203272	epithelial cell of esophagus	CTCF	epithelium, esophagus	78.83452
chr14:80203154..80203630	A549	CTCF	lung	27.89855
chr14:80203011..80203254	H1	CTCF	embryo	176.59827
chr14:80202899..80203355	esophagus squamous [illegible]	CTCF	epithelium, esophagus	36.35915
chr14:80202900..80203330	A549	CTCF	lung	47.57056
chr14:80202945..80203301	smooth muscle cell	CTCF	musculature of body	41.75899
chr14:80203024..80203240	HepG2	RAD21	endocrine gland, exocrine gland, liver, epithelium	107.77481
chr14:80203008..80203393	Panc1	CTCF	pancreas, epithelium	166.02022
chr14:80202988..80203264	AG09309	CTCF	skin of body, connective tissue	24.99974
chr14:80202833..80203349	NCI-H929	CTCF	bone marrow, bone element	43.55206
chr14:80203043..80203519	colonic mucosa	CTCF	colon, large intestine, intestine	12.84678
chr14:80202922..80203402	Calu3	CTCF		20.24922
chr14:80202921..80203345	K562	CTCF	bodily fluid, blood	31.54374
chr14:80202994..80203252	HCT116	CTCF	colon, large intestine, epithelium, intestine	67.21222
chr14:80203030..80203249	BE2C	CTCF	brain	217.82900
chr14:80202970..80203305	PC-3	CTCF	prostate gland	183.91696
chr14:80202917..80203406	OCI-LY7	CTCF	bodily fluid, blood	250.28374
chr14:80202947..80203290	DOHH2	CTCF		190.63256

chr14:80202932..80203332	liver	RAD21	endocrine gland, exocrine gland, liver	48.06698
chr14:80202935..80203340	A549	CTCF	lung	141.13291
chr14:80202898..80203428	HCT116	CTCF	colon, large intestine, epithelium, intestine	50.41569
chr14:80203003..80203262	LNCAP	CTCF	prostate gland	218.73608
chr14:80202954..80203281	HCT116	CTCF	colon, large intestine, epithelium, intestine	115.99994
chr14:80203031..80203615	HCT116	CTCF	colon, large intestine, epithelium, intestine	36.31748
chr14:80202900..80203320	right lobe of liver	CTCF	endocrine gland, exocrine gland, liver	24.59116
chr14:80203047..80203277	A549	CTCF	lung	21.54666
chr14:80202759..80203475	CD14-positive monocyte	CTCF	bodily fluid, blood	28.49304
chr14:80203004..80203253	esophagus squamous [illegible]	CTCF	epithelium, esophagus	166.53639
chr14:80202961..80203297	OCI-LY3	CTCF	lymph node, bone marrow, immune organ, bone element	169.80144
chr14:80203028..80203237	epithelial cell of proximal tubule	CTCF	kidney, epithelium	118.15048
chr14:80202945..80203325	endothelial cell of umbilical vein	CTCF	blood vessel, vein, placenta, extraembryonic component, vasculature, epithelium	55.03574
chr14:80202986..80203250	cardiac fibroblast	CTCF	heart, connective tissue	41.35428

Table S4. Expression quantitative trait loci (eQTL) data obtained for rs4794067 by Regulome DB.

**Method**	**QTL location**	**Biosample**	**Target genes**
eQTLs	chr17:47731461..47731462	lower leg skin	NPEPPS
eQTLs	chr17:47731461..47731462	lower leg skin	TBX21
eQTLs	chr17:47731461..47731462	venous blood	TBKBP1
eQTLs	chr17:47731461..47731462	gastrocnemius medialis	EFCAB13
eQTLs	chr17:47731461..47731462	testis	NPEPPS
eQTLs	chr17:47731461..47731462	testis	TBKBP1
eQTLs	chr17:47731461..47731462	omental fat pad	TBKBP1
eQTLs	chr17:47731461..47731462	thyroid gland	NPEPPS
eQTLs	chr17:47731461..47731462	esophagus squamous epithelium	EFCAB13

eQTLs	chr17:47731461..47731462	esophagus squamous epithelium	MRPL45P2
eQTLs	chr17:47731461..47731462	ascending aorta	NPEPPS
eQTLs	chr17:47731461..47731462	subcutaneous adipose tissue	TBKBP1
eQTLs	chr17:47731461..47731462	subcutaneous adipose tissue	EFCAB13
eQTLs	chr17:47731461..47731462	esophagus muscularis mucosa	NPEPPS
eQTLs	chr17:47731461..47731462	suprapubic skin	TBKBP1
eQTLs	chr17:47731461..47731462	upper lobe of left lung	TBKBP1
eQTLs	chr17:47731461..47731462	cerebellum	NPEPPS
eQTLs	chr17:47731461..47731462	pituitary gland	NPEPPS
eQTLs	chr17:47731461..47731462	tibial artery	EFCAB13
eQTLs	chr17:47731461..47731462	body of pancreas	EFCAB13
eQTLs	chr17:47731461..47731462	thyroid gland	TBKBP1
eQTLs	chr17:47731461..47731462	tibial nerve	MRPL45P2
eQTLs	chr17:47731461..47731462	tibial nerve	EFCAB13
eQTLs	chr17:47731461..47731462	tibial nerve	TBKBP1
eQTLs	chr17:47731461..47731462	tibial nerve	NPEPPS
eQTLs	chr17:47731461..47731462	ascending aorta	TBKBP1
eQTLs	chr17:47731461..47731462	omental fat pad	NPEPPS
eQTLs	chr17:47731461..47731462	esophagus squamous epithelium	NPEPPS
eQTLs	chr17:47731461..47731462	subcutaneous adipose tissue	NPEPPS
eQTLs	chr17:47731461..47731462	testis	EFCAB13
eQTLs	chr17:47731461..47731462	esophagus muscularis mucosa	TBKBP1
eQTLs	chr17:47731461..47731462	esophagus muscularis mucosa	EFCAB13
eQTLs	chr17:47731461..47731462	upper lobe of left lung	EFCAB13
eQTLs	chr17:47731461..47731462	stomach	EFCAB13
eQTLs	chr17:47731461..47731462	suprapubic skin	NPEPPS
eQTLs	chr17:47731461..47731462	gastroesophageal sphincter	EFCAB13

eQTLs	chr17:47731461..47731462	fibroblast derived cell line	TBKBP1
eQTLs	chr17:47731461..47731462	fibroblast derived cell line	MRPL45P2
eQTLs	chr17:47731461..47731462	fibroblast derived cell line	EFCAB13
eQTLs	chr17:47731461..47731462	lower leg skin	EFCAB13
eQTLs	chr17:47731461..47731462	lower leg skin	TBKBP1
eQTLs	chr17:47731461..47731462	lower leg skin	MRPL45P2
eQTLs	chr17:47731461..47731462	sigmoid colon	TBKBP1
eQTLs	chr17:47731461..47731462	tibial artery	NPEPPS
eQTLs	chr17:47731461..47731462	tibial artery	TBKBP1
eQTLs	chr17:47731461..47731462	venous blood	NPEPPS
eQTLs	chr17:47731461..47731462	gastrocnemius medialis	MRPL45P2
eQTLs	chr17:47731461..47731462	breast epithelium	NPEPPS
eQTLs	chr17:47731461..47731462	breast epithelium	EFCAB13

Table S5. Histone modification analysis of SNPs using RegulomeDB.

	**Chromatin state**	**Biosample**	**Classification**	**Organ**
rs225014	Weak Repressed PolyComb	liver	tissue	endocrine gland, exocrine gland, liver
	Weak Repressed PolyComb	liver	tissue	endocrine gland, exocrine gland, liver
rs10865710	Active enhancer 1	HuH-7	cell line	endocrine gland, exocrine gland, liver, epithelium
	Active enhancer 1	HuH-7.5	cell line	endocrine gland, exocrine gland, liver, epithelium
	Active enhancer 1	hepatic stellate cell	primary cell	endocrine gland, exocrine gland, liver, connective tissue
	Quiescent/Low	Liver	tissue	endocrine gland, exocrine gland, liver
	Quiescent/Low	Hepatocyte	in vitro differentiated cells	endocrine gland, exocrine gland, liver, epithelium

Quiescent/Low	Liver	tissue	endocrine gland, exocrine gland, liver
Quiescent/Low	Liver	tissue	endocrine gland, exocrine gland, liver
Quiescent/Low	Liver	tissue	endocrine gland, exocrine gland, liver
Quiescent/Low	Liver	tissue	endocrine gland, exocrine gland, liver
Quiescent/Low	Liver	tissue	endocrine gland, exocrine gland, liver
Quiescent/Low	Hepatocyte	primary cell	endocrine gland, exocrine gland, liver, epithelium
Quiescent/Low	right lobe of liver	tissue	endocrine gland, exocrine gland, liver
Quiescent/Low	Liver	tissue	endocrine gland, exocrine gland, liver
Weak transcription	HepG2	cell line	endocrine gland, exocrine gland, liver, epithelium
Active TSS	Liver	tissue	endocrine gland, exocrine gland, liver
Active TSS	Hepatocyte	in vitro differentiated cells	endocrine gland, exocrine gland, liver, epithelium
Active TSS	hepatic stellate cell	primary cell	endocrine gland, exocrine gland, liver, connective tissue
Active TSS	Liver	tissue	endocrine gland, exocrine gland, liver
Active TSS	Liver	tissue	endocrine gland, exocrine gland, liver
Active TSS	HepG2	cell line	endocrine gland, exocrine gland, liver, epithelium
Active TSS	Liver	tissue	endocrine gland, exocrine gland, liver
Active TSS	right lobe of liver	tissue	endocrine gland, exocrine gland, liver
Flanking TSS upstream	HuH-7	cell line	endocrine gland, exocrine gland, liver, epithelium
Flanking TSS upstream	Liver	tissue	endocrine gland, exocrine gland, liver
Flanking TSS upstream	Liver	tissue	endocrine gland, exocrine gland, liver

	Flanking TSS upstream	Hepatocyte	primary cell	endocrine gland, exocrine gland, liver, epithelium
	Flanking TSS upstream	HuH-7.5	cell line	endocrine gland, exocrine gland, liver, epithelium
	Flanking TSS upstream	Liver	tissue	endocrine gland, exocrine gland, liver
rs4794067	Bivalent Enhancer	Hepatocyte	primary cell	endocrine gland, exocrine gland, liver, epithelium
	Bivalent Enhancer	HuH-7.5	cell line	endocrine gland, exocrine gland, liver, epithelium
	Bivalent Enhancer	HuH-7	cell line	endocrine gland, exocrine gland, liver, epithelium
	Repressed PolyComb	Hepatocyte	in vitro differentiated cells	endocrine gland, exocrine gland, liver, epithelium
	Repressed PolyComb	HepG2	cell line	endocrine gland, exocrine gland, liver, epithelium
	Bivalent Enhancer	hepatic stellate cell	primary cell	endocrine gland, exocrine gland, liver, connective tissue
	Repressed PolyComb	right lobe of liver	tissue	endocrine gland, exocrine gland, liver
	Repressed PolyComb	Liver	tissue	endocrine gland, exocrine gland, liver
	Repressed PolyComb	Liver	tissue	endocrine gland, exocrine gland, liver
	Weak Repressed PolyComb	Liver	tissue	endocrine gland, exocrine gland, liver
	Weak Repressed PolyComb	Liver	tissue	endocrine gland, exocrine gland, liver
	Weak Repressed PolyComb	Liver	tissue	endocrine gland, exocrine gland, liver
	Weak Repressed PolyComb	Liver	tissue	endocrine gland, exocrine gland, liver
	Weak Repressed PolyComb	Liver	tissue	endocrine gland, exocrine gland, liver
rs12031994	Quiescent/Low	HuH-7	cell line	endocrine gland, exocrine gland, liver, epithelium
	Quiescent/Low	Liver	tissue	endocrine gland, exocrine gland, liver
	Quiescent/Low	Liver	tissue	endocrine gland, exocrine gland, liver

	Quiescent/Low	Hepatocyte	in vitro differentiated cells	endocrine gland, exocrine gland, liver, epithelium
	Quiescent/Low	Liver	tissue	endocrine gland, exocrine gland, liver
	Quiescent/Low	Liver	tissue	endocrine gland, exocrine gland, liver
	Quiescent/Low	Liver	tissue	endocrine gland, exocrine gland, liver
	Quiescent/Low	hepatic stellate cell	primary cell	endocrine gland, exocrine gland, liver, connective tissue
	Quiescent/Low	HepG2	cell line	endocrine gland, exocrine gland, liver, epithelium
	Quiescent/Low	Hepatocyte	primary cell	endocrine gland, exocrine gland, liver, epithelium
	Quiescent/Low	right lobe of liver	tissue	endocrine gland, exocrine gland, liver
	Quiescent/Low	Liver	tissue	endocrine gland, exocrine gland, liver
	Quiescent/Low	HuH-7.5	cell line	endocrine gland, exocrine gland, liver, epithelium
	Weak Repressed PolyComb	Liver	tissue	endocrine gland, exocrine gland, liver
rs11119982	Quiescent/Low	Liver	tissue	endocrine gland, exocrine gland, liver
	Quiescent/Low	HuH-7	cell line	endocrine gland, exocrine gland, liver, epithelium
	Quiescent/Low	Liver	tissue	endocrine gland, exocrine gland, liver
	Quiescent/Low	Liver	tissue	endocrine gland, exocrine gland, liver
	Quiescent/Low	right lobe of liver	tissue	endocrine gland, exocrine gland, liver
	Quiescent/Low	Liver	tissue	endocrine gland, exocrine gland, liver
	Quiescent/Low	Liver	tissue	endocrine gland, exocrine gland, liver
	Quiescent/Low	Hepatocyte	in vitro differentiated cells	endocrine gland, exocrine gland, liver, epithelium
	Repressed PolyComb	Liver	tissue	endocrine gland, exocrine gland, liver

	Weak Repressed PolyComb	Liver	tissue	endocrine gland, exocrine gland, liver
	Weak Repressed PolyComb	HepG2	cell line	endocrine gland, exocrine gland, liver, epithelium
	Weak Repressed PolyComb	hepatic stellate cell	primary cell	endocrine gland, exocrine gland, liver, connective tissue
	Weak Repressed PolyComb	Hepatocyte	primary cell	endocrine gland, exocrine gland, liver, epithelium
	Weak Repressed PolyComb	HuH-7.5	cell line	endocrine gland, exocrine gland, liver, epithelium

Table S6. Primers used for MALDI-TOF MassARRAY single-nucleotide polymorphism genotyping.

Gene	NCBI ID	PCR forward primer †	PCR reverse primer	Extension primer
DIO2	rs225014	ACGTTGGATGGTGAAATTGGGTGAGGATGC	ACGTTGGATGTCTTCTCCTGGGTACCATTG	**ggaag**GTGTGGTGCATGTCTCCAGT
	rs225017	ACGTTGGATGAGGATCTTTTGTCCATTCCA	ACGTTGGATGTGCACACCTAGTTCTCACTC	TACATTCTTAAACTAGTTGTCTGAA
PPARG	rs10865710	ACGTTGGATGCCTAGGGTCTTAGGATTCAC	ACGTTGGATGGCTTTTGGCATTAGATGCTG	**cctttt**CATTAGATGCTGTTTTGTCTT
	rs2016520	ACGTTGGATGTCTGGGTCTGAACGCAGATG	ACGTTGGATGATCCTCTTCCTTGTCACTGC	**atga**CCAGGCTGATGGGAACCA
ATF3	rs11119982	ACGTTGGATGGGTCATTGAGATGTTCTGGC	ACGTTGGATGTTCCCCATGGCCAATCCTTG	**ccct**GCCAATCCTTGGCCATC
AKT3	rs12031994	ACGTTGGATGGTACATAATCTACTTCCAGGC	ACGTTGGATGGTGACATAGGTTAGCGCTTA	GTCTTATTCTCTGGGTAGA
GADD45A	rs532446	ACGTTGGATGCTTTGTCCGACTAGAGTGTG	ACGTTGGATGAGTCCCCAGCATGCTTAGAC	**ccccc**GTACCACAGAAAATCCTG
	rs3783468	ACGTTGGATGGTTGCCTGATTGTGGATCTG	ACGTTGGATGAAACTAGCACATCTCGGCTG	AGCCACCCTCCTGACCC
TBX21	rs4794067	ACGTTGGATGTCCCTGTTCCTTAAGGTACG	ACGTTGGATGTTGGAGACCGGATACATGAC	CCCAACACCTTACCC

5′ tags on extension primers are marked in bold.
